# Trans-ancestral rare variant association study with machine learning-based phenotyping for metabolic dysfunction-associated steatotic liver disease

**DOI:** 10.1186/s13059-025-03518-5

**Published:** 2025-03-10

**Authors:** Robert Chen, Ben Omega Petrazzini, Áine Duffy, Ghislain Rocheleau, Daniel Jordan, Meena Bansal, Ron Do

**Affiliations:** 1 The Charles Bronfman Institute for Personalized Medicine, Icahn School of Medicine at Mount Sinai, New York, NY USA; 2 Department of Genetics and Genomic Sciences, Icahn School of Medicine at Mount Sinai, New York, NY USA; 3 Medical Scientist Training Program, Icahn School of Medicine at Mount Sinai, New York, NY USA; 4 Center for Genomic Data Analytics, Icahn School of Medicine at Mount Sinai, New York, NY USA; 5 Division of Liver Diseases, Icahn School of Medicine at Mount Sinai, New York, NY USA

**Keywords:** Machine learning, Metabolic dysfunction-associated steatotic liver disease, Genetic association studies

## Abstract

**Background:**

Genome-wide association studies (GWAS) have identified common variants associated with metabolic dysfunction-associated steatotic liver disease (MASLD). However, rare coding variant studies have been limited by phenotyping challenges and small sample sizes. We test associations of rare and ultra-rare coding variants with proton density fat fraction (PDFF) and MASLD case–control status in 736,010 participants of diverse ancestries from the UK Biobank, All of Us, and BioMe and performed a trans-ancestral meta-analysis. We then developed models to accurately predict PDFF and MASLD status in the UK Biobank and tested associations with these predicted phenotypes to increase statistical power.

**Results:**

The trans-ancestral meta-analysis with PDFF and MASLD case–control status identifies two single variants and two gene-level associations in *APOB*, *CDH5*, *MYCBP2*, and *XAB2*. Association testing with predicted phenotypes, which replicates more known genetic variants from GWAS than true phenotypes, identifies 16 single variants and 11 gene-level associations implicating 23 additional genes. Two variants were polymorphic only among African ancestry participants and several associations showed significant heterogeneity in ancestry and sex-stratified analyses. In total, we identified 27 genes, of which 3 are monogenic causes of steatosis (*APOB*, *G6PC1*, *PPARG*), 4 were previously associated with MASLD (*APOB*, *APOC3*, *INSR*, *PPARG*), and 23 had supporting clinical, experimental, and/or genetic evidence.

**Conclusions:**

Our results suggest that trans-ancestral association analyses can identify ancestry-specific rare and ultra-rare coding variants in MASLD pathogenesis. Furthermore, we demonstrate the utility of machine learning in genetic investigations of difficult-to-phenotype diseases in trans-ancestral biobanks.

**Supplementary Information:**

The online version contains supplementary material available at 10.1186/s13059-025-03518-5.

## Background

Metabolic dysfunction-associated steatotic liver disease (MASLD) and steatohepatitis (MASH) represent a global health concern and have an estimated heritability of 20% to 70% [1], suggesting a significant genetic predisposition. Recent genome-wide association studies (GWAS) have identified dozens of loci implicated in hepatic steatosis and metabolic dysfunction [2, 3]. However, most susceptibility loci are linked to common single nucleotide variants with minor allele frequencies (MAF) ≥ 0.01 and are predominantly located in non-coding regions, complicating the identification of the causal gene. Further, these variants explain only 20% of the heritability of MASLD [2]. In contrast, rare (MAF < 0.01) and ultra-rare (MAF < 0.0001) missense or protein-truncating variants (PTVs) may directly impact protein function, elucidate the roles of their respective genes in MASLD pathophysiology, and unveil new therapeutic targets. This is crucial given that the FDA has only approved one drug for the treatment of MASLD [4].

Exome sequencing has enabled the investigation of rare and ultra-rare coding variants across all protein-coding genes in the human genome. However, existing rare variant studies of MASLD have been relatively small, focused on European or Asian populations, and identified only five missense variants and one PTV with nominally significant associations [5–7]. Accurate phenotyping of MASLD is another challenge: in electronic health record-linked biobanks, the ICD-10 codes commonly used to define MASLD underestimate its prevalence by 42–45% [8]. While hepatic fat quantification using magnetic resonance imaging (MRI)-estimated proton density fat fraction (PDFF) addresses underdiagnosis, the requirement for specialized equipment precludes assessment in large populations: fewer than 10% of UK Biobank participants have PDFF measurements available. Further, neither approach captures the severity of the disease, including the stage of fibrosis or the presence of cirrhosis.

In this study, we first performed the largest rare variant association study of MASLD to date, including 736,010 diverse ancestry participants from the UK Biobank, *All of Us*, and Bio*Me*.

Similar to prior work using machine learning-based phenotyping to assist genetic discovery [9–14], including rare variant discovery in coronary artery disease [15], we then constructed a machine learning model to accurately predict MASLD and PDFF in the UK Biobank. We validated the predicted phenotypes by assessing their identification of known genetic variants from GWAS and used them to identify novel rare and ultra-rare coding variants. To assess genetic contributions to MASLD severity, we also examined associations with the LiverRisk score, an accurate predictor of liver stiffness previously validated in the UK Biobank [16]. For all identified genes, we curated supporting clinical, experimental, and genetic evidence and discussed their plausible mechanistic links with MASLD.

## Results

### Trans-ancestral analysis of true MASLD and PDFF in the UK Biobank, *All of Us*, and Bio*Me*

**Fig. 1:**
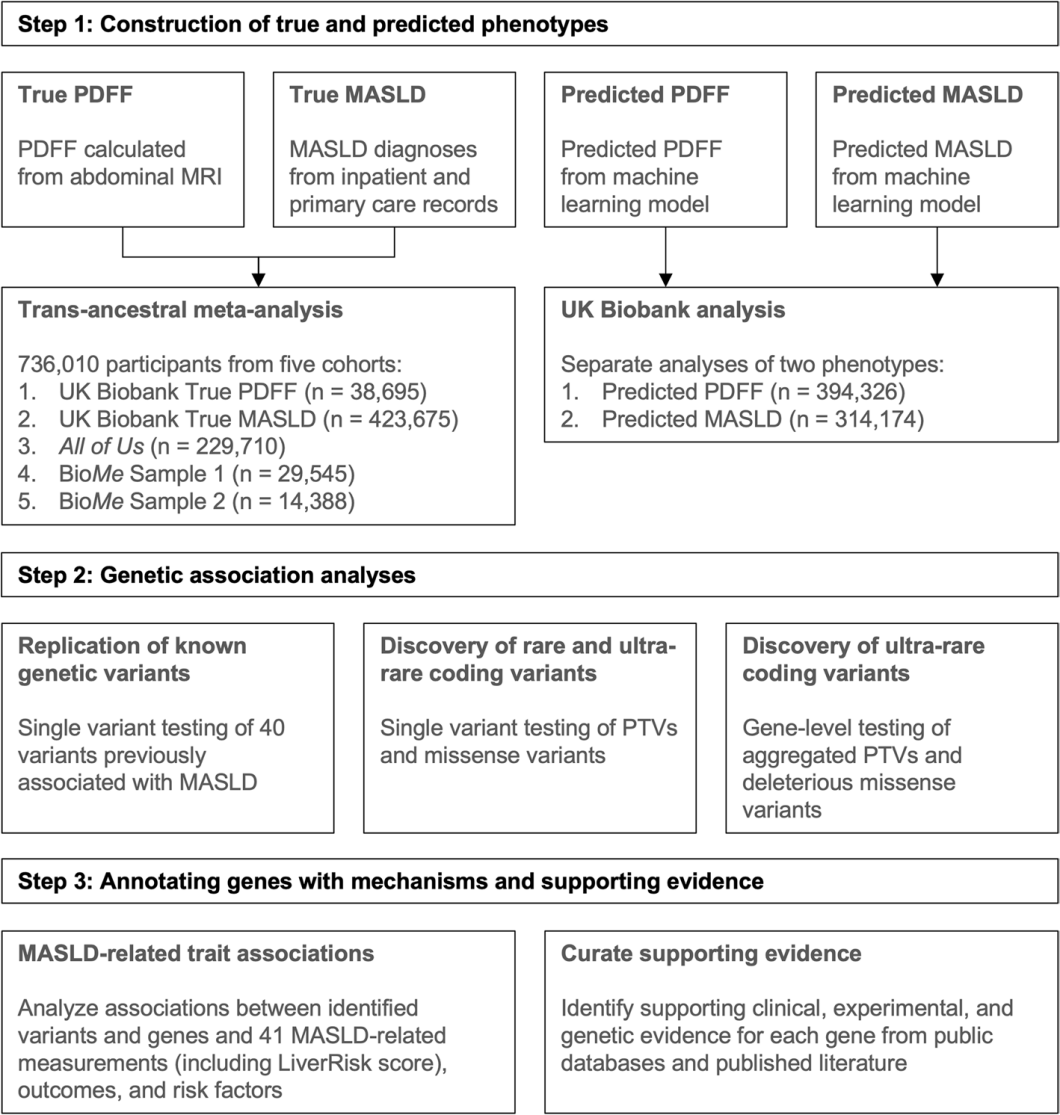
Study workflow. Abbreviations: PDFF, proton density fat fraction; MRI, magnetic resonance imaging; PTV, protein-truncating variant

Similar to the GOLDPlus GWAS meta-analysis of MASLD [2], we tested rare variant associations with PDFF and MASLD diagnoses among 736,010 participants of diverse ancestries from five cohorts and performed a meta-analysis (Fig. 1). The first cohort included UK Biobank participants with PDFF measurements (*n* = 38,695), who had a median PDFF of 2.87 (IQR 2.01–4.83), and of whom 8107 (21.0%) had a PDFF ≥ 5.5%. The other four cohorts included participants from the UK Biobank without PDFF measurements (*n* = 423,675 with 6336 (1.5%) MASLD cases), *All of Us* (*n* = 229,710 with 8504 (3.7%) MASLD cases), Bio*Me* Sample 1 (*n* = 29,545 with 864 (2.9%) MASLD cases), and Bio*Me* Sample 2 (*n* = 14,388 with 849 (5.9%) MASLD cases) (Additional file 1: Table S1). Across the five cohorts, 546,699 (74.3%) participants were assigned to European (EUR) ancestry, 73,225 (9.9%) to African (AFR) ancestry, 53,520 (7.3%) to Latino/Admixed American (AMR) ancestry, 12,907 (1.8%) to Central/South Asian (SAS) ancestry, 9404 (1.3%) to East Asian (EAS) ancestry, and 2374 (0.3%) to Middle Eastern (MID) ancestry. We did not observe the inflation of test statistics of any cohort in the meta-analysis (Additional file 2: Fig. S1a–b). All five cohorts identified two common coding variants in *PNPLA3* (rs738408 and rs738409) at genome-wide significance (Additional file 1: Table S2), validating the accurate phenotyping of MASLD (Additional file 1: Table S3).

**Fig. 2:**
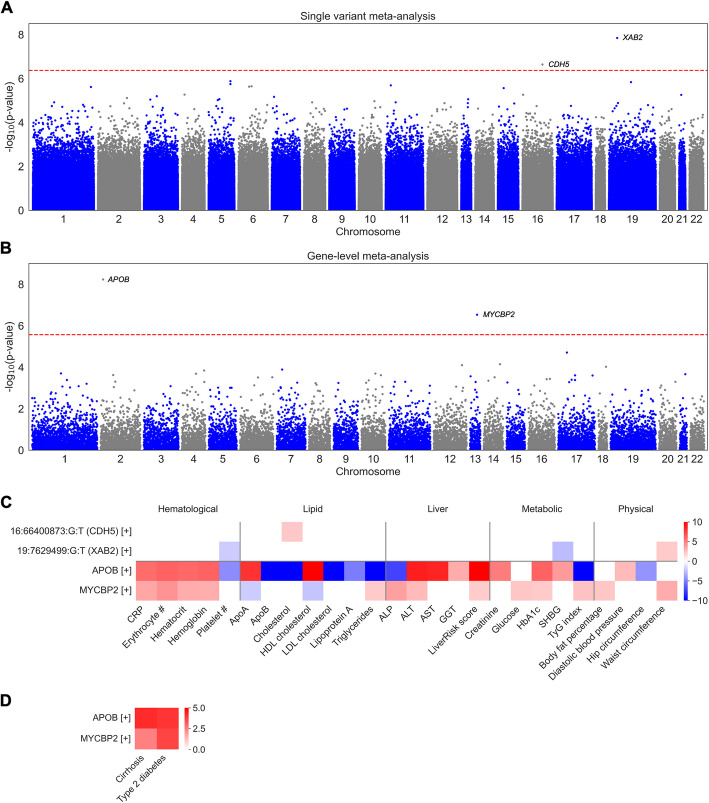
Variants and genes identified by true phenotypes across the UK Biobank, *All of Us*, and Bio*Me.* **A**–**B** Manhattan plots showing associations from single variant testing (**A**) and gene-level testing (**B**). The red dashed lines represent thresholds for exome-wide significance (**A**; 4.3 × 10^−7^) and Bonferroni significance (**B**; 2.7 × 10^−6^). **C**–**D** Single variant associations, labeled as chromosome:position:reference allele:effect allele (gene) (effect direction), and gene-level associations, labeled as gene (effect direction), with MASLD-related measurements (**C**) as well as outcomes and risk factors (**D**). Only associations with *p* < 0.05 are shown. For most measurements and all outcomes and risk factors, associations represent a meta-analysis of results from UK Biobank, *All of Us*, and Bio*Me*. The color of each box indicates the effect size and direction (*Z* score); for display purposes, *Z* scores are capped at 10 and − 10. Complete data are available in Additional file 1: Tables S9–S10. Abbreviations: CRP, C-reactive protein; ALP alkaline phosphatase; ALT, alanine aminotransferase; AST, aspartate aminotransferase; GGT, gamma-glutamyltransferase; SHBG, sex hormone binding globulin

In the meta-analysis, single variant testing of rare or ultra-rare PTVs and missense variants yielded two exome-wide significant variants (Fig. 2a; Additional file 2: Fig. S2a–b; Additional file 1: Table S4). Both were novel and neither had significant heterogeneity between cohorts: 19:7,629,499:G:T (*XAB2* p.Pro10His; *Z* = 5.67, *p* = 1.42 × 10^−8^) and 16:66,400,873:G:T (*CDH5* p.Arg565Leu; *Z* = 5.18, *p* = 2.28 × 10^−7^). In ancestry-stratified analyses, 19:7,629,499:G:T (*XAB2*) was testable and exome-wide significant only among EUR ancestry participants, while 16:66,400,873:G:T (*CDH5*) was sub-exome-wide significant among EUR ancestry participants (*p* = 3.76 × 10^−6^) but had a consistent effect among AMR ancestry participants (Additional file 1: Table S4). In gnomAD, both variants were polymorphic in EUR and AMR ancestry groups while 16:66,400,873:G:T (*CDH5*) was additionally polymorphic in the SAS ancestry group, all with allele frequencies between 1.00 × 10^−4^ and 1.10 × 10^−5^ (Additional file 1: Table S5). Neither variant exhibited significant effect heterogeneity by sex (Additional file 1: Table S4).

Gene-level testing of ultra-rare PTVs and deleterious missense variants yielded two genes with Bonferroni-significant tests in the meta-analysis: *APOB* (*Z* = 5.82, *p* = 5.90 × 10^−9^), which was previously identified in a smaller UK Biobank sample [17], and *MYCBP2* (*Z* = 4.87, *p* = 1.13 × 10^−6^) (Fig. 2b; Additional file 2: Fig. S3a–b; Additional file 1: Table S6). *APOB* had significant heterogeneity between cohorts, although it was a risk gene in all cohorts, while *MYCBP2* did not. In ancestry-stratified analyses, while both genes were Bonferroni-significant only among EUR ancestry participants, *APOB* had a consistent effect in AFR, AMR, EUR, and EAS ancestry participants without significant heterogeneity, while *MYCBP2* had significant heterogeneity between AFR and EUR ancestry participants (p_het_ = 0.04) (Additional file 1: Table S6). Neither gene exhibited significant effect heterogeneity by sex (Additional file 1: Table S6).

Previous studies have identified at least six MASLD-associated coding variants with MAF ≤ 0.01: one study of 301 MASLD cases with either advanced fibrosis or hepatocellular carcinoma identified two variants in *ATG7* [5]; a second study of a four-generation family with progressive MASLD identified a variant in *MTTP* [6]; a third study of 492 MASLD cases identified a variant in *PNPLA3* and another in *MTTP* [7]; and a fourth study of a single individual identified a variant in *GCKR* (Additional file 1: Table S7) [18]. Here, we replicated only 22:43,946,300:A:G (*PNPLA3* p.Asn455Ser; *Z* = 2.27, *p* = 0.023). We also identified 16 additional rare and ultra-rare coding variant associations with the four genes at nominal significance (Additional file 1: Table S8). The two most significant associations were 4:99,608,826:C:T (*MTTP* p.Ile564Thr; *Z* = 3.96, *p* = 7.53 × 10^−5^) and 4:99,622,756:G:T (*MTTP* p.Gly865Ter; *Z* = 3.67, *p* = 2.46 × 10^−4^), which have conflicting and pathogenic ClinVar classifications, respectively; both variants are linked to multiple cases of abetalipoproteinemia and steatosis [19–21].

The variants and genes we identified were also associated with MASLD-related measurements, risk factors, and outcomes across the UK Biobank, *All of Us*, and Bio*Me* (Fig. 2c–d; Additional file 1: Tables S9–S10). To facilitate interpretation, we henceforth refer to variants and genes positively associated with MASLD as “risk” and those negatively associated with MASLD as “protective.” From single variant testing, the risk variant 19:7,629,499:G:T (*XAB2*) was positively associated with waist circumference and negatively associated with sex hormone binding globulin (SHBG), with low SHBG being a marker of hepatic steatosis and metabolic syndrome [22]. Additionally, the risk variant 16:66,400,873:G:T (*CDH5*) was positively associated with serum cholesterol. From gene-level testing, the risk genes *APOB* and *MYCBP2* were positively associated with serum liver enzymes, LiverRisk score, cirrhosis, and type 2 diabetes. *APOB* was also negatively associated with serum lipids (apoB, LDL cholesterol, and triglycerides), consistent with loss of *APOB* function causing defective lipoprotein secretion and hepatic lipid accumulation [23].

### Construction of predicted MASLD and PDFF phenotypes in the UK Biobank

**Fig. 3:**
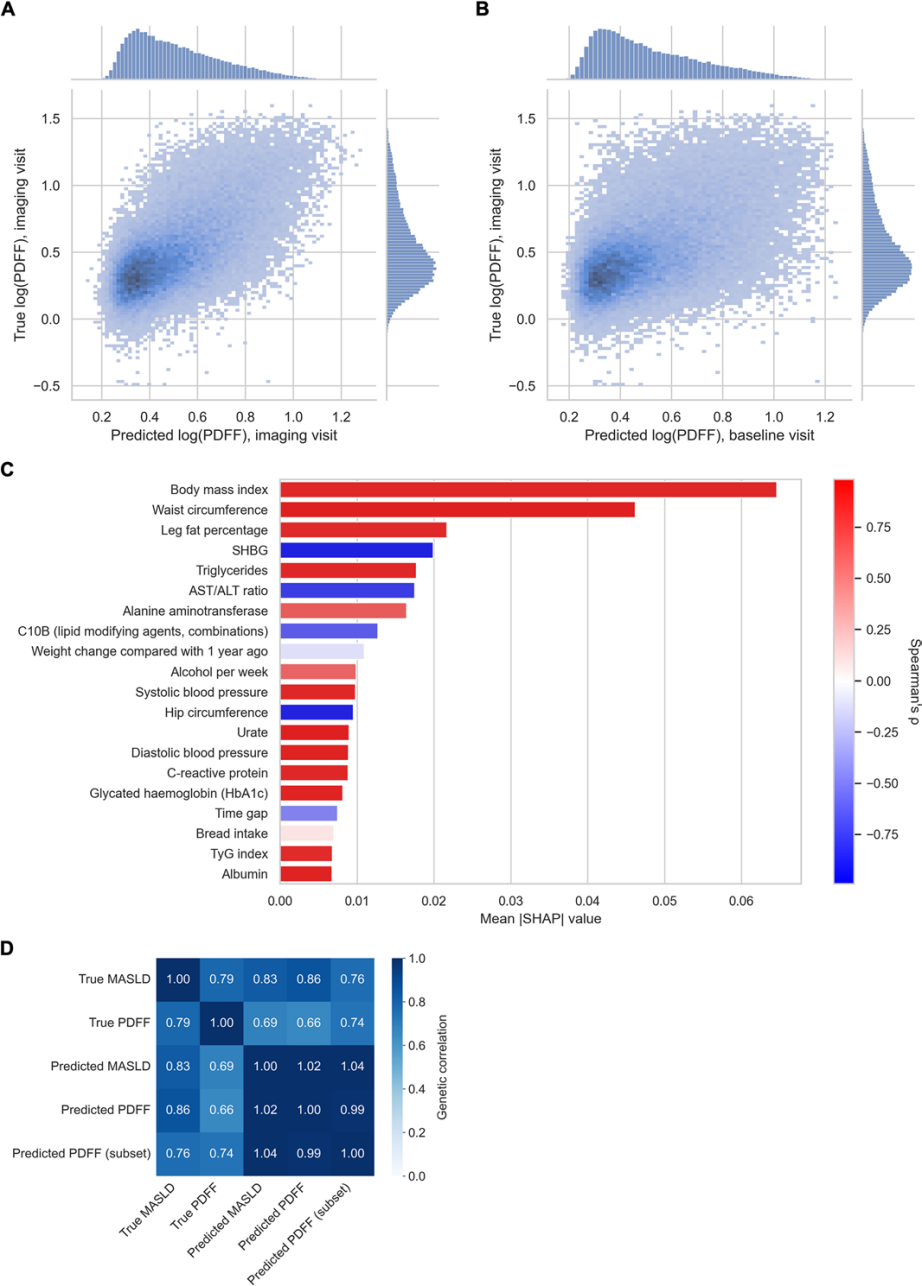
Construction of machine learning-predicted phenotypes in the UK Biobank. **A** 2D histogram comparing predicted log(PDFF) at the imaging visit with true log(PDFF) at the imaging visit. **B** 2D histogram comparing predicted log(PDFF) at the baseline visit with true log(PDFF) at the imaging visit. **C** Top 20 features for the PDFF prediction model as determined by SHapley Additive exPlanations (SHAP) analysis. The color of each bar reflects Spearman’s *ρ* between feature values and SHAP values. **D** Genetic correlations between true and predicted phenotypes in the UK Biobank. “Predicted PDFF (subset)” represents testing of predicted PDFF only among participants with true PDFF measureemnts. Abbreviations: SHBG (sex hormone binding globulin)

Given the limited discoveries from the true phenotype analysis, we aimed to increase power in genetic association testing using predicted phenotypes (Fig. 1). We constructed machine learning models using 183 features representing comprehensive phenotypic data to predict log(PDFF) among 38,876 UK Biobank participants with PDFF measurements (Additional file 1: Tables S1, S11). Except for laboratory measurements, which were obtained pre-imaging, all features were obtained at the time of imaging. In holdout testing for all participants, model predictions attained *r* = 0.66 (95% CI 0.65–0.66) and *R*^2^ = 0.43 (95% CI 0.42–0.44) with true log(PDFF) measurements (Fig. 3a). Model predictions also attained an area under the receiver operating characteristic curve (AUROC) of 0.86 (95% CI 0.86–0.87) and area under the precision-recall curve (AUPRC) of 0.64 (95% CI 0.63–0.65) in identifying steatosis (PDFF ≥ 5.5%). For AUROC, *r*, and *R*^2^, there was better performance among participants with shorter time gaps between laboratory measurements and PDFF imaging; however, these differences were not substantial (Additional file 1: Table S12).

We used trained models to generate PDFF predictions for 418,695 UK Biobank participants (Additional file 1: Table S1), using values from the baseline visit for all features. Among 38,485 of these participants who had predictions at both the baseline and imaging timepoints, there was high correlation between the two predictions (*r* = 0.87 (95% CI 0.87–0.88), *R*^2^ = 0.72 (95% CI 0.71–0.72)) and between the baseline prediction and PDFF at the imaging timepoint (*r* = 0.53 (95% CI 0.53–0.54), *R*^2^ = 0.26 (95% CI 0.24–0.27)) (Fig. 3b), despite a median time gap of 10.1 years (interquartile range (IQR) 8.8–11.8). To predict MASLD status, we stratified the 418,695 participants into 257,796 likely controls and 76,409 likely cases using predicted PDFF thresholds of ≤ 4% (negative predictive value = 0.92) and ≥ 6% (positive predictive value = 0.71), respectively (Additional file 1: Table S13).

A SHAP analysis demonstrated that the most important features of the model were consistent with known MASLD risk factors and mechanisms. The most important features (highest mean absolute SHAP value) were body mass index (BMI), waist circumference, leg fat percentage, SHBG, triglycerides, and AST/ALT ratio (Fig. 3c; Additional file 1: Table S14). We also examined correlations between feature values and SHAP values to assess the direction of the effect of each feature; SHBG and AST/ALT ratio had negative correlations, while the other four top features had positive correlations.

We then used these phenotypes for genetic association testing in the UK Biobank (Fig. 1; Additional file 1: Table S1). For predicted MASLD, we analyzed 314,174 participants consisting of 72,175 predicted cases and 241,999 predicted controls. For predicted PDFF, we analyzed 394,326 participants with a median predicted PDFF of 3.34 (IQR 2.41–5.16), of whom 86,825 (22.0%) had a predicted PDFF ≥ 5.5%. These represented 9- and tenfold increases in effective sample sizes compared to true MASLD and true PDFF, respectively, and power calculations suggested that predicted phenotypes would yield increased power to varying degrees across multiple fixed values of explained variation (Additional file 1: Table S15).

### Common variant associations in known MASLD genes in the UK Biobank

In the UK Biobank, we performed genome-wide association testing of the MASLD/PDFF phenotypes and found high genetic correlations between true MASLD and both predicted MASLD (*r*_*g*_ = 0.83) and predicted PDFF (*r*_*g*_ = 0.86), supporting the accuracy of the predicted phenotypes (Fig. 3d). True and predicted PDFF were also highly genetically correlated (*r*_*g*_ = 0.74) when analyzing the same participant subset (i.e., those with PDFF measurements). True and predicted PDFF had similar heritability estimates (*h*^2^ = 0.129 and 0.124, respectively), but predicted MASLD had significantly higher heritability compared to true MASLD (*h*^2^ = 0.044 and 0.003, respectively) likely due to the identification of undiagnosed cases (Additional file 1: Table S16).

We examined 40 variants associated with MASLD in published GWASs (Additional file 1: Table S17), including 16 linkage disequilibrium-independent variants reported in the GOLDPlus meta-analysis [2]. Predicted MASLD and predicted PDFF replicated 16 and 18 of the 40 variants at genome-wide significance and 29 and 30 of these variants at nominal significance, respectively. In comparison, true MASLD, true PDFF, and a meta-analysis of these two true phenotypes identified only 6, 13, and 15 variants at genome-wide significance and 22, 23, and 24 variants at nominal significance, respectively. Effect sizes and directions were generally consistent between true and predicted phenotypes (Additional file 2: Fig. S4). Association testing with the predicted phenotypes yielded smaller *p*-values for 30 of the variants compared to the true phenotype meta-analysis, likely due to increased sample size of predicted compared to true PDFF.

Because some MASLD-associated genes exhibit sexual dimorphism [24, 25], we further performed sex-stratified analyses, with predicted PDFF replicating an additional variant in *TMC4/MBOAT7* at nominal significance among women only (Additional file 1: Table S18). Additionally, four variants in *GPAM*, *TM6SF2*, *APOE*, and *PNPLA3* had significant heterogeneity between men and women for both predicted and true phenotypes, although effect directions were consistent. Finally, *COBLL1* is known to have stronger effects on fasting glucose and obesity in women compared to men [24], and consistent with this, 2:164,699,029:A:G (*COBLL1*) had a larger effect in women (*β* = − 0.048, SE = 0.003, *p* = 3.71 × 10^−73^) compared to men (*β* = − 0.019, SE = 0.003, *p* = 9.83 × 10^−14^) for predicted PDFF with significant heterogeneity (*p* = 2.17 × 10^−15^).

At genome-wide significance, we replicated a total of 24 variants in 19 genes. Variants in *APOE*, *CHUK*, *COBLL1*, *CWF19L1*, *ERLIN1*, *GCKR*, *PNPLA2*, *PNPLA3*, *TM6SF2*, and *TRIB1* were replicated by both true and predicted phenotypes; variants in *FTO*, *INSR*, and *SREBF1* were replicated only by the predicted phenotypes; and variants in *ADH1B*, *GPAM*, *MARC1*, *MTTP*, *TMC4*/*MBOAT7*, and *TOR1B* were replicated only by the true phenotypes. For 19 of these variants that were replicated by multiple phenotypes, including 15 identified by both true and predicted phenotypes, effect directions were consistent across all phenotypes. Together, these results suggest the predicted phenotypes can accurately identify genetic associations with MASLD with greater statistical power compared to the true phenotypes.

### Rare and ultra-rare coding variant associations using predicted phenotypes in the UK Biobank

**Fig. 4:**
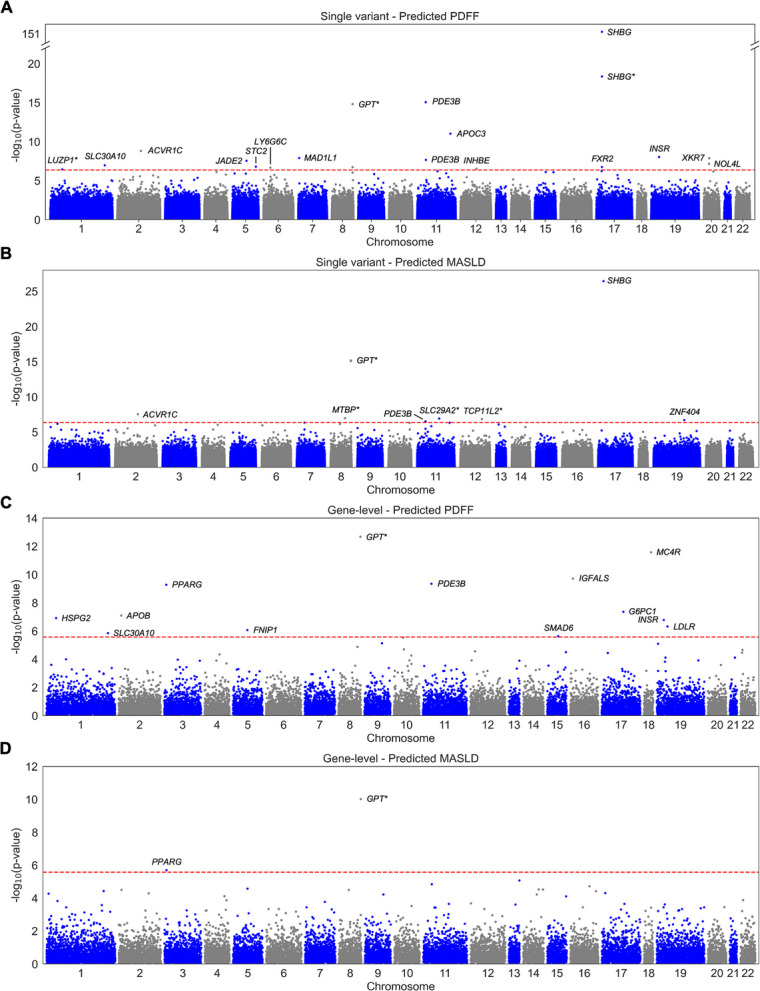
Variants and genes identified by predicted phenotypes in the UK Biobank. **A**–**B** Manhattan plots showing associations from single variant testing (**A**) and gene-level testing (**B**). The red dashed lines represent thresholds for exome-wide significance (**A**; 4.3 × 10^−7^) and Bonferroni significance (**B**; 2.7 × 10^−6^). Genes and variants marked with an asterisk did not pass post hoc filtering (i.e., nominal association with a true phenotype or with both liver enzymes and metabolic dysfunction markers) and were not further analyzed

We next performed rare and ultra-rare variant association testing for predicted MASLD and predicted PDFF (Fig. 4a–d) and did not observe inflation of test statistics (Additional file 2: Fig. S5). As predictions were imperfect, we anticipated that some variants and genes might show significant associations with the predicted phenotypes without being causal for MASLD; for example, three variants in *GPT* (which encodes ALT) were negatively associated with both serum ALT and predicted MASLD/PDFF (Additional file 1: Tables S9, S19), but variants causing isolated changes in liver enzymes do not cause metabolic dysfunction [26]. Conversely, other variants may cause metabolic dysfunction without affecting hepatic function. Therefore, although we report all significant associations, we prioritized variants and genes nominally associated with a true phenotype or with both liver enzymes and metabolic dysfunction markers for subsequent analyses.

From single variant testing, predicted MASLD and predicted PDFF identified 4 and 15 exome-wide significant variants from 4 and 14 genes, respectively (Additional file 1: Table S19). Between the two phenotypes, there were 16 distinct variants, of which 3 had positive associations (“risk variants”) and 13 had negative associations (“protective variants”). For predicted PDFF, the most significant variants were 17:7,631,360:C:T (*SHBG* p.Pro185Leu; *β* = 0.20, SE = 0.01, *p* = 3.97 × 10^−152^), 11:14,843,853:C:T (*PDE3B* p.Arg893Ter; *β* = − 0.16, SE = 0.02, *p* = 8.89 × 10^−16^), 11:116,830,638:G:A (*APOC3* splice donor; *β* = − 0.09, SE = 0.01, *p* = 9.55 × 10^−12^), 2:157,550,353:A:G (*ACVR1C* p.Ile195Thr; *β* = − 0.08, SE = 0.01, *p* = 1.56 × 10^−9^), and 19:7,125,507:C:T (*INSR* p.Val1012Met; *β* = − 0.04, SE = 0.01, *p* = 9.31 × 10^−9^). The variants in *SHBG*, *PDE3B*, and *ACVR1C* were also identified by predicted MASLD. Of the 16 variants, 12 had consistent effect directions with true MASLD/PDFF in the trans-ancestral meta-analysis, and effect sizes were similar when comparing true and predicted phenotypes in the UK Biobank (Additional file 2: Fig. S6). Further, six variants had nominally significant associations with true MASLD/PDFF in either the trans-ancestral meta-analysis or a component cohort: 2:157,550,353:A:G (*ACVR1C*; *p* = 0.009), 6:31,719,166:C:T (*LY6G6C* p.Arg103Gln; *p* = 0.029), 11:14,843,853:C:T (*PDE3B*; *p* = 0.011), 11:116,830,638:G:A (*APOC3*; *p* = 0.003), 17:7,593,064:C:T (*FXR2* p.Arg483Gln; *p* = 0.005), 17:7,631,360:C:T (*SHBG*; *p* = 9.69 × 10^−5^). These variants had consistent effect directions between true and predicted phenotypes except for 11:116,830,638:G:A (*APOC3*); however, this variant had significant heterogeneity in the trans-ancestral meta-analysis, and the association between *APOC3* and MASLD is known to differ between populations [27].

From gene-level testing, predicted MASLD and predicted PDFF identified 12 distinct genes with Bonferroni-significant tests, respectively (Additional file 1: Table S20), of which 5 had positive associations (“risk genes”) and 7 had negative associations (“protective genes”). Predicted PDFF identified all 12 genes: *APOB* (*β* = 0.12, SE = 0.02, *p* = 8.12 × 10^−8^), *FNIP1* (*β* = − 0.43, SE = 0.09, *p* = 8.71 × 10^−7^), *G6PC1* (*β* = 0.13, SE = 0.02, *p* = 4.37 × 10^−8^), *HSPG2* (*β* = − 0.07, SE = 0.02, *p* = 1.22 × 10^−7^), *IGFALS* (*β* = 0.10, SE = 0.02, *p* = 1.92 × 10^−10^), *INSR* (*β* = − 0.13, SE = 0.03, *p* = 1.67 × 10^−7^), *LDLR* (*β* = − 0.09, SE = 0.02, *p* = 4.86 × 10^−7^), *MC4R* (*β* = − 0.14, SE = 0.02, *p* = 2.62 × 10^−12^), *PDE3B* (*β* = − 0.12, SE = 0.02, *p* = 4.53 × 10^−10^), *PPARG* (*β* = 0.22, SE = 0.04, *p* = 5.29 × 10^−10^), *SLC30A10* (*β* = − 3.81 × 10^−4^, SE = 0.04, *p* = 1.43 × 10^−6^), and *SMAD6* (*β* = − 0.07, SE = 0.02, *p* = 2.35 × 10^−6^). Predicted MASLD also identified *PPARG* (*β* = 1.36, SE = 0.28, *p* = 2.03 × 10^−6^). Of the 12 genes, *APOB* was also Bonferroni-significant in the trans-ancestral meta-analysis (*p* = 5.90 × 10^−9^), while 7 additional genes were nominally significant in either the meta-analysis or a component cohort: *G6PC1* (*p* = 0.002), *HSPG2* (*p* = 0.035), *IGFALS* (*p* = 0.011), *INSR* (*p* = 0.009), *PDE3B* (*p* = 0.007), *SLC30A10* (*p* = 0.025), and *SMAD6* (*p* = 0.007). Effect directions were consistent between true and predicted phenotypes for all genes except for *SLC30A10*.

Ancestry-stratified analyses demonstrated that these results were primarily driven by EUR ancestry participants, who comprise 87% of the UK Biobank (Additional file 1: Table S1). However, most variants were polymorphic in the AFR, AMR, and EUR ancestry groups in gnomAD (Additional file 1: Table S5). Notably, two variants were polymorphic and exome-wide significant only among AFR ancestry participants (1:219,915,459:G:A (*SLC30A10*; *p* = 1.79 × 10^−7^), 7:2,069,319:C:T (*MAD1L1*; *p* = 3.43 × 10^−7^)) (Additional file 2: Fig. S7), and both variants had higher gnomAD allele frequencies in AFR compared to EUR ancestry participants (Additional file 1: Table S5). Another six variants were nominally significant in a non-EUR ancestry, none of which had significant heterogeneity across ancestries: 17:7,631,360:C:T (*SHBG*) among AMR (*p* = 0.014), 19:7,125,507:C:T (*INSR*) among SAS (*p* = 0.020), 20:31,996,867:A:T (*XKR7*) among AFR (*p* = 3.39 × 10^−4^), and 20:32,453,712:C:T (*NOL4L*) among AFR (*p* = 3.26 × 10^−5^) (Additional file 1: Table S21). At the gene level, three genes (*PDE3B*, *SLC30A10*, *SMAD6*) had significant heterogeneity across ancestries, while all other genes did not (Additional file 1: Table S21). *SLC30A10* was Bonferroni-significant among AFR ancestry participants (*p* = 1.79 × 10^−7^), while three other genes were nominally significant in a non-European ancestry: *HSPG2* among AMR ancestry participants (*p* = 0.011), *INSR* among EAS ancestry participants (*p* = 4.96 × 10^−3^), and *SMAD6* among AFR ancestry participants (*p* = 0.030) (Additional file 2: Fig. S8). These results demonstrate the value of including non-EUR ancestry participants even in a UK Biobank-restricted analysis.

Sex-stratified analyses demonstrated effect heterogeneity by sex for four single variants (1:219,915,459:G:A (*SLC30A10*), 11:14,843,853:C:T (*PDE3B*), 17:7,631,360:C:T (*SHBG*), 19:43,873,681:C:T (*ZNF404*)) and three gene-level associations (*INSR*, *LDLR*, *PPARG*), although effect directions were consistent in all cases (Additional file 1: Table S22). Effect sizes were larger in women compared to men for all these variants and genes except *LDLR*, where there was a larger effect size for men. Supporting these findings, sexual dimorphism in the expression or function of all these genes except *ZNF404* and *INSR* has previously been reported [28–33].

**Fig. 5:**
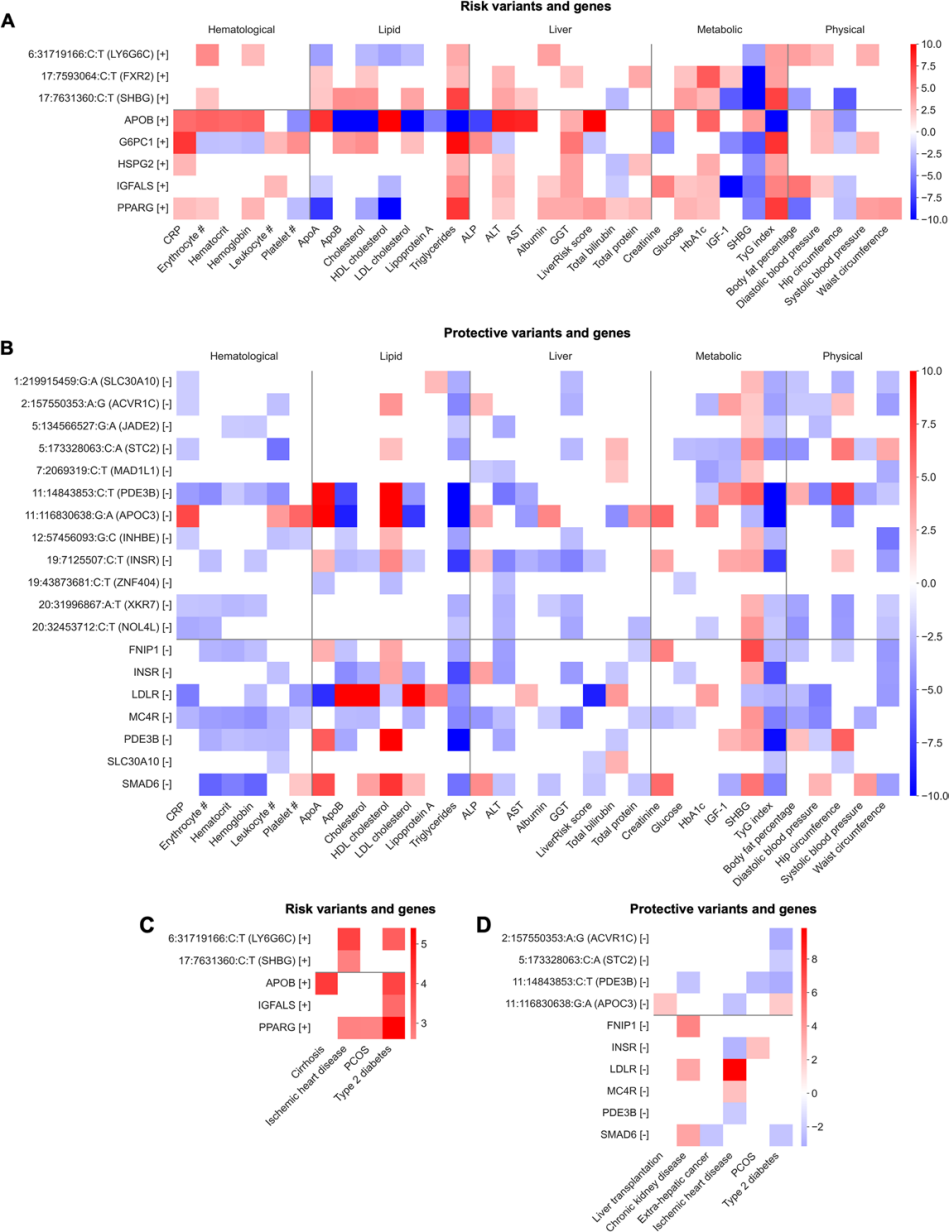
MASLD-related trait associations for predicted phenotype variants and genes. **A**–**D** Single variant associations, labeled as chromosome:position:reference allele:effect allele (gene) (effect direction), and gene-level associations, labeled as gene (effect direction), with MASLD-related measurements (**A**–**B**) as well as outcomes and risk factors (**C**–**D**). Only associations with *p* < 0.05 are shown. For most phenotypes, associations represent a meta-analysis of results from UK Biobank, *All of Us*, and Bio*Me*. The color of each box indicates the effect size and direction (*Z* score). Complete data are available in Additional file 1: Tables S9–S10. **A**,** C** Single variants and genes positively associated with predicted MASLD/PDFF. **B**,** D** Single variants and genes negatively associated with predicted MASLD/PDFF. Abbreviations: CRP, C-reactive protein; ALP, alkaline phosphatase; ALT, alanine aminotransferase; AST, aspartate aminotransferase; GGT, gamma-glutamyltransferase; SHBG, sex hormone binding globulin

Across the UK Biobank, *All of Us*, and Bio*Me*, risk variants and genes had simultaneous positive associations with liver enzymes, insulin resistance markers (HbA1c, IGF-1, and TyG index), serum lipids, and physical measurements (Fig. 5a; Additional file 1: Table S9), whereas protective variants and genes had the inverse (Fig. 5b). Conversely, most risk variants and genes were negatively associated with SHBG, while most protective variants and genes were positively associated. Risk variants and genes also had positive associations with ischemic heart disease, polycystic ovary syndrome, and type 2 diabetes (Fig. 5c; Additional file 1: Table S10), while five protective variants and one gene were negatively associated with type 2 diabetes (Fig. 5d). Finally, *APOB*, *PPARG*, *INSR*, *LDLR*, *SLC30A10*, and *SMAD6* had associations with LiverRisk score in the same effect direction as predicted MASLD/PDFF (Fig. 5a–b). Together, these results suggest that these variants and genes have concurrent roles in hepatic inflammation, hepatic fibrosis, and metabolic dysfunction.

### Functional and clinical annotations of single variants

We obtained functional and ClinVar annotations for all 18 single variants: 2 from true phenotypes and 16 from predicted phenotypes (Additional file 1: Table S23). Three of the variants are PTVs: 11:14,843,853:C:T (*PDE3B*) is a stop gain variant expected to cause nonsense-mediated decay, while 11:116,830,638:G:A (*APOC3*) and 12:57,456,093:G:C (*INHBE*) are splice donor and splice acceptor variants with SpliceAI scores of 0.89 and 0.98, respectively. Of the 15 missense variants, 2 are predicted by AlphaMissense as “likely pathogenic” and 4 have REVEL scores > 0.5. Six variants were reported in ClinVar: two classified as benign or likely benign, two as variants of uncertain significance, and two with conflicting classifications (Additional file 1: Table S23).

### Evidence supporting variants and genes

**Fig. 6:**
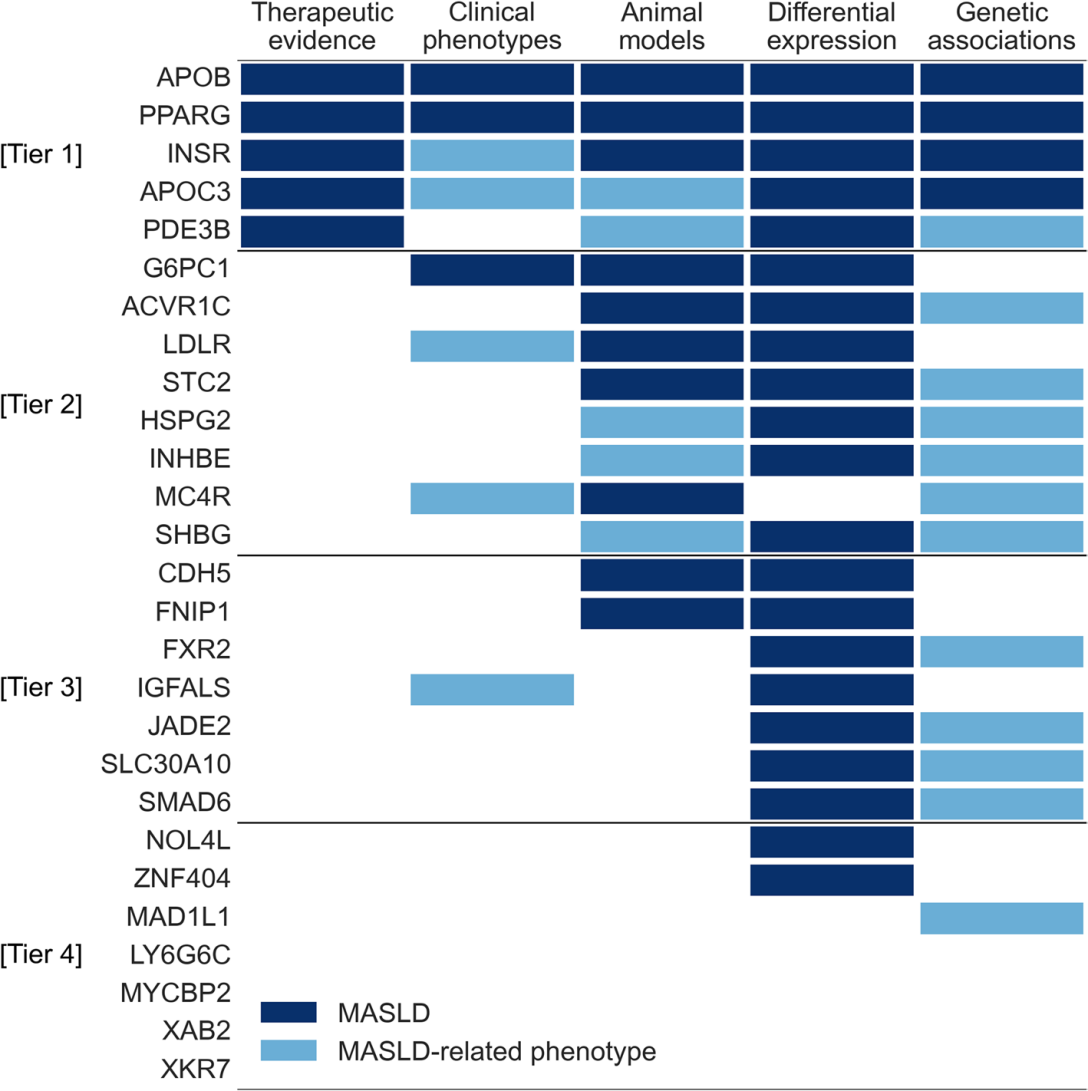
Supporting evidence for MASLD-associated genes. Clinical, experimental, and genetic evidence for genes identified in this study. Genes in tiers 1, 2, 3, and 4 have ≥ 4, 3, 2, or ≤ 1 sources of evidence, respectively. For clinical phenotypes, “MASLD” includes monogenic causes of steatosis (e.g., abetalipoproteinemia, familial partial lipodystrophy, glycogen storage disease 1a). For clinical phenotypes, animal models, and genetic associations, “MASLD-related phenotype” includes metabolic dysfunction phenotypes (e.g., metabolic syndrome, type 2 diabetes). Complete data are available in Additional file 1: Table S24

We categorized the 27 distinct genes from this study—4 genes in the trans-ancestral meta-analysis and an additional 23 genes from the predicted phenotype analyses—into four tiers based on supporting clinical, experimental, and genetic evidence (Fig. 6). Specifically, 5 genes (*APOB*, *INSR*, *PPARG*, *APOC3*, *PDE3B*) have human therapeutic evidence, 8 genes cause monogenic diseases that present with MASLD and/or metabolic dysfunction, 15 had animal model evidence, and 21 genes were differentially expressed in MASLD compared to control hepatocytes (Additional file 1: Table S24). Additionally, 4 genes (*APOB*, *INSR*, *PPARG*, *APOC3*) had prior genetic associations with MASLD while another 12 genes had associations with a MASLD-related phenotype (type 2 diabetes, BMI-adjusted waist-to-hip ratio (WHRadjBMI), metabolic syndrome). In total, 23 of the 27 genes had at least one source of supporting evidence.

## Discussion

This study assessed the relationship between rare and ultra-rare coding variants and several MASLD-related phenotypes. Identification of these variants has been limited by small sample sizes, as fewer than 2% and 10% of UK Biobank participants have clinical MASLD diagnoses and PDFF measurements, respectively. It is also unknown whether these variants affect fibrosis development. Here, we first performed a trans-ancestral meta-analysis of five cohorts that included 10,217 MASLD cases from outside the UK Biobank, identifying significant associations in one known gene (*APOB*) and three novel genes (*CDH5*, *MYCBP2*, *XAB2*). We next performed association testing on 314,174 and 394,326 UK Biobank participants with accurately predicted MASLD and PDFF phenotypes, respectively. This identified significant associations in an additional 23 genes, with three associations reaching exome-wide/Bonferroni significance only among AFR ancestry participants. Supporting the feasibility of increasing statistical power through phenotype prediction, we demonstrated that of 40 known common variants previously discovered from GWAS, predicted phenotypes replicated more variants than true phenotypes at both genome-wide and nominal significance. Third, using the LiverRisk score as a proxy for liver fibrosis, we identified several genes (*APOB*, *INSR*, *LDLR*, *MYCBP2*, *PPARG*, *SLC30A10*, *SMAD6*) that likely have concurrent roles in steatosis and fibrosis. Finally, we found that 23 of the 27 genes we identified have supporting clinical, experimental, and/or genetic evidence linking them to MASLD.

The three novel genes we identified in the meta-analysis have plausible roles in MASLD. *CDH5* encodes VE-cadherin, a known MASLD biomarker whose expression is reduced by insulin [34, 35]. Decreased VE-cadherin expression causes hepatic vascular endothelial dysfunction and steatosis, but this may be countered by the natural product erianin [35]. *MYCBP2* inhibits signaling pathways that drive hepatic steatosis, including mTOR and p38 MAPK [36–39], while also suppressing adipogenesis by promoting SMAD4 ubiquitination [40], possibly explaining why aggregated deleterious variants in *MYCBP2* were positively associated with both MASLD and body fat percentage. *XAB2* encodes a splicing factor that is required for ERCC1-XPF and XPG-mediated R-loop processing [41]. Aberrant R-loop processing leads to cellular inflammation and senescence [42], both of which are implicated in MASLD, and liver-specific inactivation of *XPG* results in senescence-induced fat accumulation [43]. Further, defective ERCC1-XPF causes increased GLUT1 and GLUT3-mediated hepatocyte glucose uptake, and the resulting hyperglycemia activates pro-inflammatory responses via mTOR [44].

Several genes we identified are involved in lipid metabolism, including *APOB*, *APOC3*, *LDLR*, and *PPARG*. First, *APOB* LOF variants cause familial hypobetalipoproteinemia, wherein defective hepatic lipoprotein secretion results in hepatic steatosis that frequently progresses to fibrosis and hepatocellular carcinoma [17, 23, 45, 46]. Accordingly, aggregated ultra-rare variants in *APOB* were positively associated with both true and predicted MASLD/PDFF phenotypes, LiverRisk score, and cirrhosis, but negatively associated with serum apoB, cholesterol, and ischemic heart disease. Second, although associations between *APOC3* and MASLD are unclear, prior studies have only identified common variants that increase MASLD risk [27]. Here, a rare splice donor *APOC3* variant (11:116,830,638:G:A) known to lower triglycerides and increase HDL cholesterol is also negatively associated with predicted PDFF and serum AST (*p* = 6.98 × 10^−5^) [47, 48]. This protective effect is consistent with *APOC3* inhibition via volanesorsen reducing hepatic steatosis in patients with hypertriglyceridemia [49], and substantial evidence suggests *APOC3* deficiency, unlike *APOB* deficiency, is cardioprotective without causing steatosis [50–52]. Mechanistically, stable isotope studies in humans suggest that *APOC3* deficiency promotes VLDL lipolysis by lipoprotein lipase and conversion to LDL without increasing hepatic uptake of VLDL remnants [53], ultimately reducing the lipid load available for the liver. Third, aggregated ultra-rare variants in *LDLR* were negatively associated with predicted PDFF but positively associated with serum cholesterol and ischemic heart disease. These variants also had a non-significant negative association with true PDFF (*p* = 0.053). LDL receptor defects cause familial hypercholesterolemia, suggesting these are LOF variants that decrease hepatic lipoprotein uptake at the expense of increased cardiovascular risk [54]. Finally, *PPARG* protects against MASLD, *PPARG* deficiency causes familial partial lipodystrophy 3 that presents with steatosis [55], and PPAR-γ agonists like pioglitazone are under clinical investigation for MASLD [56]. Here, we identified a positive relationship between aggregated ultra-rare variants in *PPARG* and predicted PDFF.

Several variants and genes have been associated with WHRadjBMI, with larger ratios suggesting unhealthy adiposity and insulin resistance and vice versa. LOF variants in *INSR*, *ACVR1C* (including 2:157,550,353:A:G), *PDE3B* (including 11:14,843,853:C:T), and *INHBE* were associated with decreased WHRadjBMI [57–59], while a *SMAD6* variant was associated with increased WHRadjBMI [60]. Common *INSR* variants were also associated with MASLD in the GOLDPlus meta-analysis [2]. Here, we found that rare variants in *INSR*, *ACVR1C*, *PDE3B*, and *INHBE*, as well as aggregated ultra-rare variants in *PDE3B*, *INSR*, and *SMAD6*, were negatively associated with predicted PDFF. Supporting these associations, three variants in *ACVR1C* and *PDE3B*, two of which are predicted LOF, as well as aggregated ultra-rare variants in *INSR*, *PDE3B*, and *SMAD6*, had nominally significant associations with true MASLD/PDFF in the same direction. Further, mice deficient in *ACVR1C*, *PDE3B*, or *INHBE* show decreased body weight gain, favorable fat distribution profiles, and increased insulin sensitivity [61–64]. However, the mechanism by which *INSR* variants may protect against MASLD is unclear. One explanation is that, while patients homozygous for *INSR* LOF variants present with severe insulin resistance [65], those heterozygous are generally asymptomatic, and heterozygous impairment of INSR expression protects from and reverses diet-induced hepatic steatosis in mice [66]. Alternatively, these may be gain-of-function variants with similar effects to INSR overexpression [67], which improves obese and diabetic phenotypes in mice. Regardless, the variants we tested were not significantly associated with type 2 diabetes and were negatively associated with liver enzymes, serum lipids, and glucose, supporting a protective effect.

We replicated clinical associations of *G6PC1*, *IGFALS*, *SHBG*, and *SLC30A10* with MASLD and MASLD-related phenotypes. First, aggregated ultra-rare variants in *G6PC1* were positively associated with true PDFF; this is consistent with *G6PC1* deficiency causing glycogen storage disease type 1a, which presents with hyperlipidemia and hepatic steatosis [68, 69]. Delivering functional *G6PC1* may ameliorate steatosis in patients with deficiency [70]. Second, reduced insulin-like growth factor-1 (IGF-1) levels are associated with increased histological severity of MASLD [71]; here, we found that aggregated ultra-rare *IGFALS* variants were positively associated with predicted PDFF and negatively associated with serum IGF-1. This is consistent with the product of *IGFALS* (acid-labile subunit) increasing the serum half-life of IGF-1 [72], and patients with *IGFALS* deficiency present with IGF-1 deficiency and insulin resistance [73]. Third, low SHBG causes metabolic dysfunction via mechanisms dependent on and independent of sex hormone transport [22]; here, we identified a rare variant (17:7,631,360:C:T) positively associated with predicted PDFF and negatively associated with serum SHBG. Although SHBG was a feature of the PDFF prediction model, this variant was associated with true PDFF in the same direction with *p* = 9.69 × 10^−5^. Fourth, *SLC30A10* deficiency causes hypermanganesemia and liver cirrhosis in humans [74], and LOF variants are associated with MRI cT1, a marker of steatohepatitis and fibrosis [75, 76]. In contrast, we found an ultra-rare *SLC30A10* variant (1:219,915,459:G:A) that was negatively associated with predicted PDFF, triglycerides, GGT, and body fat percentage, suggesting a possible gain-of-function protective effect. This variant was polymorphic only among AFR ancestry participants, consistent with known differential selection of zinc transporter genes in AFR compared to other ancestries [77].

Finally, three genes (*STC2*, *FNIP1*, and *MC4R*) have been associated with MASLD in mouse models. First, overexpression of *STC2* improved hepatic steatosis and hypertriglyceridemia in obese mice [78]; here, we identified a rare variant (5:173,328,063:C:A) negatively associated with predicted PDFF and serum triglycerides. Second, mice deficient in *FNIP1* are resistant to diet-induced hepatic steatosis; consistent with this, aggregated ultra-rare variants in *FNIP1* were negatively associated with predicted PDFF [79]. Third, although *MC4R* deficiency causes steatosis via hyperphagia and obesity [80, 81], we found that aggregated ultra-rare variants in *MC4R* were negatively associated with predicted PDFF. Consistent with a gain-of-function effect, these variants were also negatively associated with serum lipids, liver enzymes, and body fat percentage. This may reflect our inclusion of BMI as a covariate to identify variants with BMI-independent effects, and gain-of-function variants in *MC4R* are known to have BMI-independent protective effects against obesity and type 2 diabetes [82, 83].

Only one drug (resmetirom) is FDA-approved to treat MASLD [4], and it is indicated for moderate/advanced fibrosis. For most patients, management continues to consist of dieting, exercise, and treatment of concomitant type 2 diabetes. Thus, our results may be useful for informing therapeutic development, including from a precision medicine approach for patients with variants in our identified genes. For example, the nonselective phosphodiesterase inhibitor pentoxifylline has been shown to reduce steatosis and fibrosis in MASLD patients [84]; this activity may be mediated through PDE3B inhibition. Additionally, PPARγ agonism via pioglitazone improves both steatosis and fibrosis in MASH patients [85–88]. Finally, four other genes we identified (*ACVR1C*, *CDH5*, *INHBE*, *STC2*) are under preclinical investigation [35, 49, 59, 78, 89]. However, our results also suggest caution with therapeutics that reduce hepatic lipoprotein secretion: aggregated ultra-rare *APOB* variants that reduced serum apoB were positively associated with both MASLD and fibrosis, and a phase 3 study of an apoB synthesis inhibitor (mipomersen) resulted in increased steatosis and liver enzymes in several patients [90, 91].

In the study, we investigated whether variants conferring risk to MASLD have ancestry or sex-specific effects. Only one of the GOLDPlus meta-analysis loci showed significant heterogeneity by ancestry and none by sex [2]. Similarly, we found no significant heterogeneity by ancestry for any of the single variants, and only three single variants in *SLC30A10*, *PDE3B*, and *SHBG* had significant heterogeneity by sex. Among gene-level associations, we observed significant heterogeneity by ancestry for four genes (*MYCBP2*, *PDE3B*, *SLC30A10*, *SMAD6*) and significant heterogeneity by sex for three genes (*INSR*, *LDLR*, *PPARG*). However, heterogeneity in gene-level associations likely reflects different variants within the same gene being tested among different participant subsets; for example, variants polymorphic among only EUR ancestry participants would not be reflected in gene-level associations for AFR ancestry participants. Such heterogeneity could also be due to small sample sizes in ancestry-specific cohorts.

Our study also demonstrates the importance of diverse phenotyping methods: each phenotype (true MASLD, true PDFF, predicted MASLD, predicted PDFF) identified variants that all other phenotypes did not, suggesting that different phenotype definitions capture different aspects of the disease, thereby enriching the understanding and detection of genetic associations. ICD-10 codes are widely available across biobanks and best reflect clinical diagnostic criteria, but they underrepresent the true prevalence of MASLD cases and misclassify cases and controls [8], reducing statistical power in genetic association testing. PDFF addresses underdiagnosis by providing a quantitative measure of steatosis, but its limited availability also limits sample sizes.

Finally, while our predicted phenotypes extended PDFF measurements to all UK Biobank participants, they were subject to error and ultimately reflected the features used for prediction.

This study has several limitations. First, our trans-ancestral meta-analysis relied on ICD-10 codes, which have limited accuracy, to define MASLD outside of the UK Biobank PDFF cohort. This was our impetus for analyzing genetic associations with predicted phenotypes, but we were unable to extend them to *All of Us* and Bio*Me* due to incomplete feature data. This limitation underscores the need for large, diverse biobanks with multimodal data. Second, most associations with both true and predicted phenotypes were driven by EUR ancestry participants, likely due to the limited numbers of participants of other ancestries. However, several associations were either exome-wide/Bonferroni-significant or nominally significant among non-EUR ancestry participants. Third, when testing genetic associations with predicted phenotypes, prediction inaccuracies may result in the identification of variants not truly associated with MASLD. To mitigate this, we prioritized variants nominally associated with true phenotypes and/or simultaneously associated with liver enzymes and metabolic dysfunction markers. Fourth, although we discussed plausible mechanistic relationships between MASLD and 18 of the 27 genes we identified, many of the remaining genes have limited functional characterization and/or are pleiotropic and require further investigation. Fifth, we did not have access to liver histology data or liver stiffness measurements, so our study could not assess the full spectrum of MASLD pathologies and relied on the LiverRisk score to predict fibrosis severity. Future large-scale studies integrating genetics with true or predicted histology could provide additional insights.

## Conclusions

In conclusion, this study aggregated MASLD phenotypes from 736,010 participants across three biobanks and used machine learning to comprehensively phenotype MASLD and PDFF among approximately 400,000 UK Biobank participants. These approaches enabled powerful rare variant association analyses that implicated 27 genes in MASLD pathogenesis, 23 of which are novel. Many of these genes, including *APOB*, *INSR*, and *PPARG*, have multiple sources of supporting evidence and are implicated in known MASLD mechanisms like lipid metabolism, insulin resistance, and adiposity. These findings provide a roadmap for future studies investigating novel genes and pathways underlying MASLD development and progression.

## Methods

### Study population

We used data from the UK Biobank, *All of Us*, and Bio*Me*. UK Biobank includes 502,411 participants from across the United Kingdom; participants aged 40–69 were enrolled starting in 2006, and follow-up data was available for these participants until September 2023. We included 501,289 of these participants with known self-reported sex and date of birth. *All of Us* consists of more than 400,000 selectively enrolled participants from across the United States. Bio*Me* consists of 57,805 selectively enrolled participants from the Mount Sinai Health System.

We examined four different phenotypes among these participants (Fig. 1). In the UK Biobank, we assessed true PDFF, true MASLD case–control status, predicted MASLD case–control status, and predicted PDFF, while in *All of Us* and Bio*Me*, we assessed only true MASLD case–control status. Similar to the GOLDPlus meta-analysis [2], we included both true PDFF and true MASLD case–control status as phenotypes in the trans-ancestral meta-analysis.

For all phenotypes, we adapted consensus guidelines for MASLD research using electronic health records [92]. For true PDFF, we used the earliest available fat-referenced PDFF measurement (field 24,352) for each participant and excluded participants with exclusionary diagnoses (Additional file 1: Table S3). For true MASLD case/control status, we identified MASLD cases in all cohorts using ICD-10 codes K75.8 and K76.0 in inpatient and primary care records, similar to the GOLDPlus meta-analysis [2]. Among cases only, we excluded those with exclusionary diagnoses and/or who did not meet cardiometabolic criteria for MASLD (Additional file 1: Table S3) [93]. In the UK Biobank, we also excluded participants in the true PDFF sample to avoid sample overlap during the meta-analysis. Construction of the predicted MASLD and predicted PDFF phenotypes are discussed in “Prediction of MASLD and PDFF.”

### Prediction of MASLD and PDFF

We constructed gradient boosting models to predict log-transformed PDFF [log(PDFF)] measurements using LightGBM (version 4.0.0). PDFF prediction models were LightGBM regression models that minimized the mean squared error between true and predicted measurements with the following parameters: {‘boosting_type’: ‘goss’, ‘num_boost_round’: 750, ‘learning_rate’: 0.01, ‘num_leaves’: 60, ‘min_data_in_leaf’: 100}. Because LightGBM performs internal feature selection during tree construction, and to avoid introducing bias, we did not perform additional feature selection [94]. Models used 183 features (Additional file 1: Table S11), including 64 laboratory measurements, 11 physical measurements, the 50 most frequent ATC codes, 25 modified Elixhauser comorbidities (Additional file 1: Table S3), 28 lifestyle factors, and 5 covariates (age, self-reported gender, fasting time, time between laboratory measurements and PDFF, and age at laboratory measurements).

Of 42,616 participants with PDFF measurements and without diagnosis exclusions, we included 38,876 participants who had at least 38 of 75 laboratory and physical measurements and who had body mass index (BMI), waist circumference, alanine aminotransferase (ALT), aspartate aminotransferase (AST), and triglyceride measurements. For all features other than laboratory measurements, we used values from the same visit as their PDFF measurement. For laboratory measurements, which were only available from pre-imaging visits, we used values from the most recent visit and accounted for the time gap as a covariate. To prioritize participants with shorter time gaps, we applied weights to participants inversely proportional to their time gap as follows: max[5, (maximum time gap)/(participant’s time gap)].

To train models, we partitioned the 38,876 participants into ten subsets, or folds, and used a nested cross-validation approach for robust model training and evaluation. In this process, each fold served as a holdout set once, while the other nine folds were further divided into ten smaller folds for training and validation. Specifically, within these nine folds, one fold was used for validation and the remaining nine for training. After model training using its designated validation set, each model was tested on the holdout set and then used to make predictions on all participants not included in the training cohort. This process was iterated ten times for each initial fold, resulting in a total of 100 iterations (10 outer folds × 10 inner folds).

For the predicted PDFF phenotype, we generated predictions using each of 100 model iterations for 418,695 participants without diagnosis exclusions, who had at least 38 of 75 laboratory and physical measurements, and who had body mass index, waist circumference, ALT, AST, and triglyceride measurements. For each participant, we removed predictions from iterations where the participant was included in the training or validation sets and averaged predictions from the remaining iterations. For all features, we used values from the baseline visit and set the time gap covariate to 0.

For the predicted MASLD phenotype, we partitioned the 418,695 participants into those with predicted PDFF ≤ 4% (predicted controls; *n* = 257,796) and those with predicted PDFF ≥ 6% (predicted cases; *n* = 76,409), excluding those with predicted PDFF between 4 and 6% to optimize negative and positive predictive values, respectively (Additional file 1: Table S13). Among the predicted cases, we removed those who did not meet cardiometabolic criteria for MASLD.

### Participant quality control for genetic association testing

In the UK Biobank, we excluded participants with chromosomal sex discordant with self-reported sex (fields 22,001 and 31, respectively), presence of sex chromosome aneuploidy (field 22,019), outliers for heterozygosity or missing rate (field 22,027), and/or ten or more third-degree relatives (field 22,021). In Bio*Me*, we excluded participants with chromosomal sex discordant with self-reported sex, high rates of heterozygosity (standard deviation ≥ 6), genotyping rate lower than 95%, and/or genotyping concordance for single nucleotide variants (SNVs) lower than 80%. In *All of Us*, we excluded participants with fingerprint discordance, sex discordant with self-reported sex at birth, call rate ≤ 98%, cross-individual contamination rate ≥ 3%, and/or who failed coverage thresholds.

### Performing genetic association testing

We used a similar methodology for participant and variant quality control and genetic association testing in all cohorts. In brief, we first performed a pooled analysis of participants of all ancestries within each cohort and meta-analyzed results across cohorts. To examine ancestry and sex-specific associations, we separately performed ancestry and sex-stratified analyses within each cohort, meta-analyzed results across cohorts for each sex, and performed a meta-meta-analysis across ancestries and sexes, respectively. For significant variants and genes, we also tested associations with laboratory and physical measurements as well as MASLD-related outcomes and risk factors (Additional file 1: Table S3).

We encoded all binary phenotypes as 0 or 1 and applied rank-inverse normal transformation to all continuous phenotypes using the R RNOmni package (version 1.0.1.2). For all genetic association testing, we included age, sex, alcohol consumption in g/week, body mass index, and 10 principal components of ancestry (field 22,009) as covariates. We calculated weekly alcohol consumption in the UK Biobank using the approach of Evangelou et al. [95], and in *All of Us* using survey data regarding drink frequency and average daily drink count; this statistic was already calculated in Bio*Me*. For association testing of significant variants with 67 of the 80 measurements that represented blood biochemistry, we included fasting time (hours since last meal; field 74) as an additional covariate [96].

Following previous pooled ancestry approaches [15, 97, 98], and because regenie controls for population structure, we pooled together individuals from all ancestries in each cohort into a single model for primary analyses of both true and predicted phenotypes. For the trans-ancestry meta-analysis of true MASLD/PDFF, we meta-analyzed pooled results across cohorts (see the “Meta-analyses of genetic associations” section). This pooled approach offers two advantages compared to the alternative approach of performing ancestry-specific meta-analyses, performing meta-analysis across cohorts within each ancestry, and then performing meta-analyses across ancestries: first, we can test ultra-rare variants that have sufficient minor allele count (MAC) to be testable in the pooled cohort but are too rare within each ancestry to be testable; and second, there is likely greater uncertainty when performing ancestry-specific testing due to the small number of MASLD cases within each ancestry, especially in *All of Us* and Bio*Me*. Nevertheless, as sensitivity analyses, we separately performed ancestry-stratified and sex-stratified analyses within each cohort.

We performed association testing using regenie (version 3.2.2) [99]; in step 1, a whole genome model is fit using a subset of available genetic markers, while in step 2, a larger set of markers is tested for association with each trait. For step 1, which is the same for all subsequent analyses, we used unimputed genotype data to generate ridge regression predictions on blocks of 2000 SNVs. Across all cohorts, we filtered genotype data for variants with MAC > 100, minor allele frequency (MAF) ≥ 0.01, genotyping rate > 0.9, and Hardy–Weinberg exact test *p*-value < 1 × 10^−15^ using PLINK (version 2.00).

We performed step 2 separately for each of three analyses: (1) replication of known genomic variants, (2) single-variant testing of rare and ultra-rare coding variants, and (3) gene-level testing of ultra-rare coding variants. In analyses 1 and 2, we performed single-variant testing of blocks of 500 SNVs, which involves Firth logistic regression models for binary phenotypes and linear regression models for continuous phenotypes. For analysis 1, we tested variants with MAF > 0.001, genotyping rate > 0.9, and Hardy–Weinberg exact test *p*-value < 1 × 10^−15^ from Haplotype Reference Consortium-imputed genotype data, including 40 variants previously associated with MASLD in published literature, and used *p* < 5 × 10^−8^ to define genome-wide significant single variant associations. For analysis 2, using exome sequencing data, we tested variants with MAC ≥ 10, MAF < 0.01, genotyping rate > 0.9, and Hardy–Weinberg exact test *p*-value < 1 × 10^−15^ that were predicted by Ensembl variant effect predictor tool (VEP; version 111) as either protein-truncating variants (PTVs; including “transcript ablation,” “splice acceptor,” “splice donor,” “stop gained,” and “frameshift” consequences) or missense variants (including “missense,” “stop lost,” “start lost,” “transcript amplification,” “inframe insertion,” “inframe deletion,” and “protein altering” consequences) in all MANE Select transcripts for protein coding genes. Following the approach of Sveinbjornsson et al. [100], we used *p* < 4.3 × 10^−7^ to define exome-wide significant single variant associations. For analysis 3, using exome sequencing data, we performed gene-level testing of aggregated ultra-rare variants (MAF < 0.0001) that Ensembl VEP predicted as either PTVs or deleterious missense variants, requiring a minimum cumulative MAC of 10 per gene. We defined deleterious missense variants as those predicted to be deleterious or protein intolerant by each of PolyPhen-2 HumVAR, PolyPhen-2 HumDIV, Sorting Intolerant from Tolerant, Likelihood Ratio Test, and MutationTaster. We then performed standard burden tests (BURDEN), sequence kernel association tests (SKAT), optimal unified SKAT (SKATO), and aggregated Cauchy association tests (ACAT) using regenie. We used a Bonferroni-corrected *p*-value threshold (0.05/number of genes tested, or 0.05/18,520) to define significant gene-level associations. When performing ancestry-stratified and sex-stratified analyses for analyses 2 and 3, we included variants and genes that had a MAC ≥ 5 but < 10 within each ancestry or sex if the variant was also included in the primary pooled analysis (i.e., had a MAC ≥ 10 among participants of all ancestries).

For analysis 1, we estimated inflation, heritability, and genetic correlation by performing performed LD score regressions using LDSC (version 1.0.1) with default parameters. We calculated LD scores using 10,000 randomly selected UK Biobank participants. For analysis 2, we estimated the proportion of phenotypic variance explained by each variant (PVE) as follows [101], where MAF is the minor allele frequency and SE is the standard error:

$$\text{PVE}=\frac{2{\beta }^{2}\times \text{MAF}\times (1-\text{MAF})}{2{\beta }^{2}\times \text{MAF}\times \left(1-\text{MAF}\right)+{\text{SE}}^{2}\times 2\text{N}\times \text{MAF}\times (1-\text{MAF})}$$

### Meta-analyses of genetic associations

For the trans-ancestral meta-analysis, we meta-analyzed pooled ancestry results from five cohorts (UK Biobank PDFF, UK Biobank MASLD, *All of Us*, Bio*Me* Sample 1, Bio*Me* Sample 2) using METAL (release 2020–05-05) [102]. For ancestry-stratified and sex-stratified analyses, we meta-analyzed ancestry-specific and sex-specific results from the five cohorts for each ancestry and sex, respectively. We then meta-analyzed ancestry-specific and sex-specific meta-analysis results.

To determine meta-analysis *p*-values, we used a sample size and direction of effect analysis, the same approach used in the GOLDPlus analysis, instead of a standard error (SE)-based analysis [2]. This was for several reasons: (1) differences in effect sizes between true PDFF and true MASLD cohorts in the UK Biobank; (2) differences in effect sizes due to different MASLD prevalences in the UK Biobank, *All of Us*, and Bio*Me*; (3) regenie *p*-values being calculated using a likelihood ratio test, causing *Z* scores to be slightly different from *β*/SE; and (4) absent effect size estimates when using SKAT, SKATO, or ACAT for gene-level testing. We analyzed effect heterogeneity using Cochran’s *Q* test and considered *Q* test *p*-values < 0.10 significant. To determine meta-analysis effect sizes (betas) and SEs, we simultaneously conducted SE-based meta-analyses, but caution that these estimates may be inaccurate due to the aforementioned reasons.

### Post hocfiltering of associations with predicted phenotypes

We performed post-hoc filtering of associations with predicted phenotypes to remove likely spurious associations. In the GOLDPlus meta-analysis, all MASLD-associated variants had simultaneous associations with liver enzymes and metabolic dysfunction markers [2]. As such, for associations with predicted MASLD and predicted PDFF, we selected variants and genes associated with at least one liver enzyme (ALP, ALT, AST, GGT) and two different metabolic dysfunction markers (apoA, apoB, body fat percentage, cholesterol (HDL, LDL, or total), glucose, HbA1c, hip circumference, IGF-1, Lp(A), SHBG, triglycerides, waist circumference).

### Identification of corroborating evidence

We identified corroborating clinical, experimental, and genetic evidence for each gene (Fig. 6; Additional file 1: Table S24). This included human therapeutic modulation data from Open Targets and the literature; clinical phenotypes from OMIM [103]; animal model data from the literature; two sets of differential gene expression data (SteatoSite and E-GEOD-37031) [104, 105]; and prior genetic associations from the GWAS Catalog (MASLD (EFO_0003095), type 2 diabetes (MONDO_0005148), and WHRadjBMI (EFO_0007788)) and a multivariate genomic analysis of metabolic syndrome [106].

## Supplementary Information

Additional file 1. Supplementary tables. Table S1: Characteristics of different participant cohorts analyzed in this study. Table S2: Genome-wide significant common variant associations in PNPLA3 with true MASLD and PDFF. Table S3: Definitions of exclusionary diagnoses, Elixhauser comorbidities, MASLD outcomes, and MASLD risk factors. Table S4: Exome-wide significant rare and ultra-rare variant associations with true MASLD and PDFF. Table S5: Single variant allele frequencies in gnomAD v4.1.0. Table S6: Bonferroni-significant gene-level associations with true MASLD and PDFF. Table S7: Previously reported rare variants associated with MASLD. Table S8: Nominally significant rare coding variant associations with true MASLD and PDFF in GCKR, ATG7, MTTP, and PNPLA3. Table S9: Nominally significant associations of identified variants with laboratory and physical measurements. Table S10: Nominally significant associations of identified genes with laboratory and physical measurements. Table S11: Features included in the machine learning model to predict PDFF. Table S12: Performance metrics for PDFF prediction models among participants with different time gaps. Table S13: Performance metrics for identifying steatosisat different thresholds of predicted PDFF. Table S14: Most important features for the PDFF prediction model. Table S15: Power estimates for true and predicted phenotypes. Table S16: Heritability of and genetic correlation between MASLD and PDFF phenotypes in the UK Biobank. Table S17: Replication of 40 previously reported MASLD-associated variants. Table S18: Replication of 40 previously reported MASLD-associated variants in sex-stratified analyses. Table S19: Exome-wide significant rare and ultra-rare variant associations with predicted MASLD and PDFF. Table S20: Bonferroni-significant gene-level associations with predicted MASLD and PDFF. Table S21: Ancestry-stratified single variant and gene-level associations with predicted MASLD and PDFF. Table S22: Sex-stratified single variant and gene-level associations with predicted MASLD and PDFF. Table S23: Functional and clinical annotations for single variants identified in this study.

Additional file 2. Supplementary figures. Fig. S1: Quantile-quantile plots for true phenotype single-variant associations. Fig. S2: Cohort-stratified, ancestry-stratified, and pooled single variant associations for true phenotypes. Fig. S3: Cohort-stratified, ancestry-stratified, and pooled gene-level associations for true phenotypes. Fig. S4: Comparison of true and predicted phenotype effects for 40 previously reported common variants. Fig. S5: Quantile-quantile plots for predicted phenotype single variant associations. Fig. S6: Comparison of true and predicted phenotype effects for variants identified by predicted phenotypes. Fig. S7: Ancestry-stratified and pooled single variant associations for predicted phenotypes. Fig. S8: Ancestry-stratified and pooled gene-level associations for predicted phenotypes.

## Data Availability

Summary statistics and analysis code supporting the conclusions of this article are available under a Creative Commons Attribution 4.0 International license in Zenodo (10.5281/zenodo.14804238) [107]. The raw datasets supporting the conclusions of this article are accessible upon application from the UK Biobank (https://www.ukbiobank.ac.uk) and from All of Us (https://allofus.nih.gov).
