# Supplementary material for: Trans-ancestral rare variant association study with machine learning-based phenotyping for metabolic dysfunction-associated steatotic liver disease
Source: Genome Biol. 2025 Mar 10;26:50. doi: 10.1186/s13059-025-03518-5 (PMC11892324; doi:10.1186/s13059-025-03518-5)
Supplement: Supplementary file 2 — Additional file 2. Supplementary figures. Fig. S1: Quantile-quantile plots for true phenotype single-variant associations. Fig. S2: Cohort-stratified, ancestry-stratified, and pooled single variant associations for true phenotypes. Fig. S3: Cohort-stratified, ancestry-stratified, and pooled gene-level associations for true phenotypes. Fig. S4: Comparison of true and predicted phenotype effects for 40 previously reported common variants. Fig. S5: Quantile-quantile plots for predicted phenotype single variant associations. Fig. S6: Comparison of true and predicted phenotype effects for variants identified by predicted phenotypes. Fig. S7: Ancestry-stratified and pooled single variant associations for predicted phenotypes. Fig. S8: Ancestry-stratified and pooled gene-level associations for predicted phenotypes. [file 13059_2025_3518_MOESM2_ESM.docx]

**Additional file 2: Supplementary figures**

This file contains the following figures:

Fig. S1: Quantile-quantile plots for true phenotype single-variant associations

Fig. S2: Cohort-stratified, ancestry-stratified, and pooled single variant associations for true phenotypes

Fig. S3: Cohort-stratified, ancestry-stratified, and pooled gene-level associations for true phenotypes

Fig. S4: Comparison of true and predicted phenotype effects for 40 previously reported common variants

Fig. S5: Quantile-quantile plots for predicted phenotype single variant associations

Fig. S6: Comparison of true and predicted phenotype effects for variants identified by predicted phenotypes

Fig. S7: Ancestry-stratified and pooled single variant associations for predicted phenotypes

Fig. S8: Ancestry-stratified and pooled gene-level associations for predicted phenotypes

**Fig. S1: Quantile-quantile plots for true phenotype single-variant associations**

**
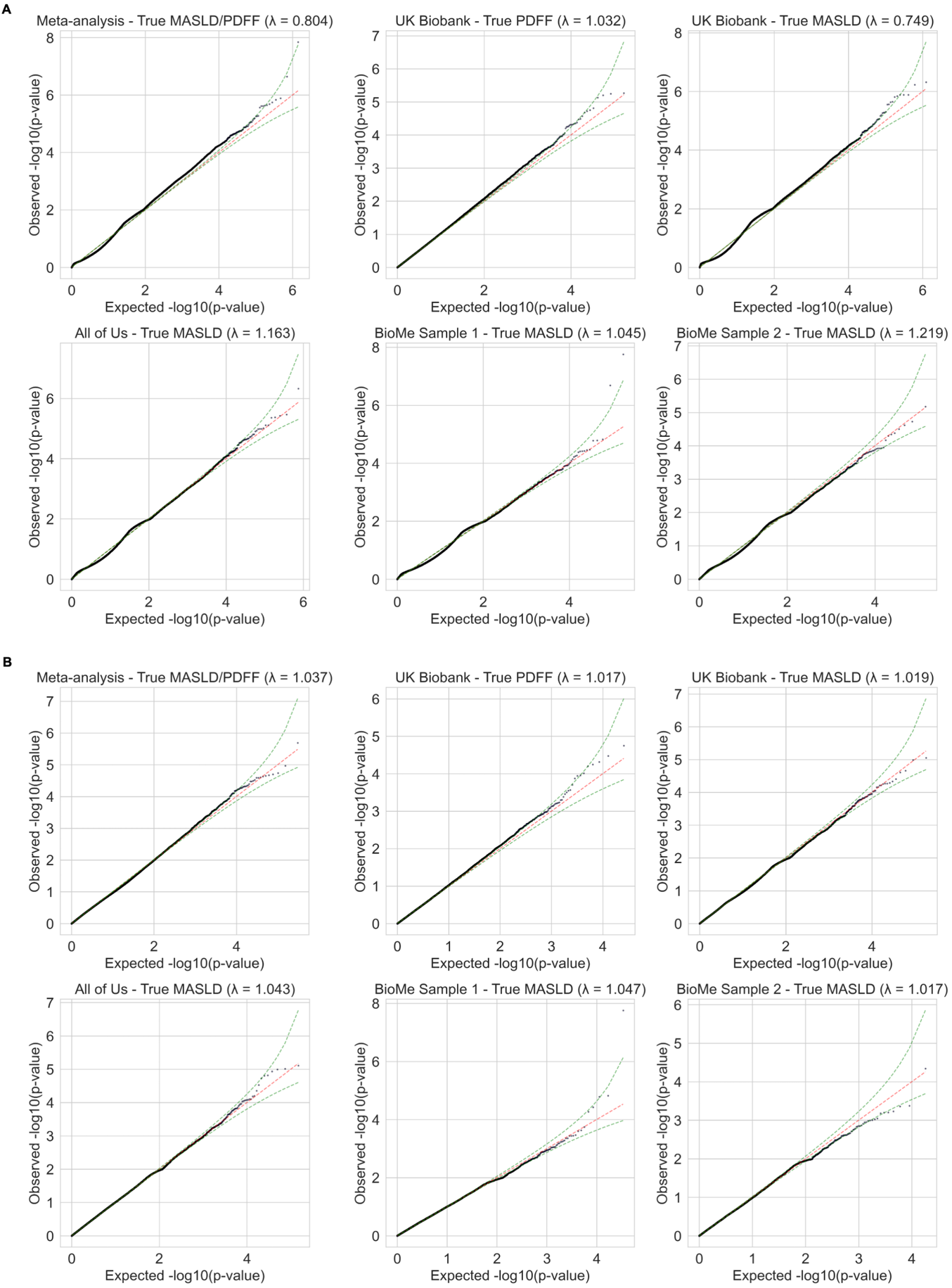
**

**(A)** Quantile-quantile plots for all tested variants [minor allele count (MAC) ≥ 10 in each cohort]. **(B)** Quantile-quantile plots only for variants with MAC ≥ 100 in each cohort. Note that the test statistic deflation in the meta-analysis and the “UK Biobank - True MASLD” cohort observed in **(A)** is no longer present when restricting to variants with MAC ≥ 100 in **(B)**. Green dashed lines represent the 95% confidence interval for expected -log_10_(p-values).

**Fig. S2: Cohort-stratified, ancestry-stratified, and pooled single variant associations for true phenotypes**
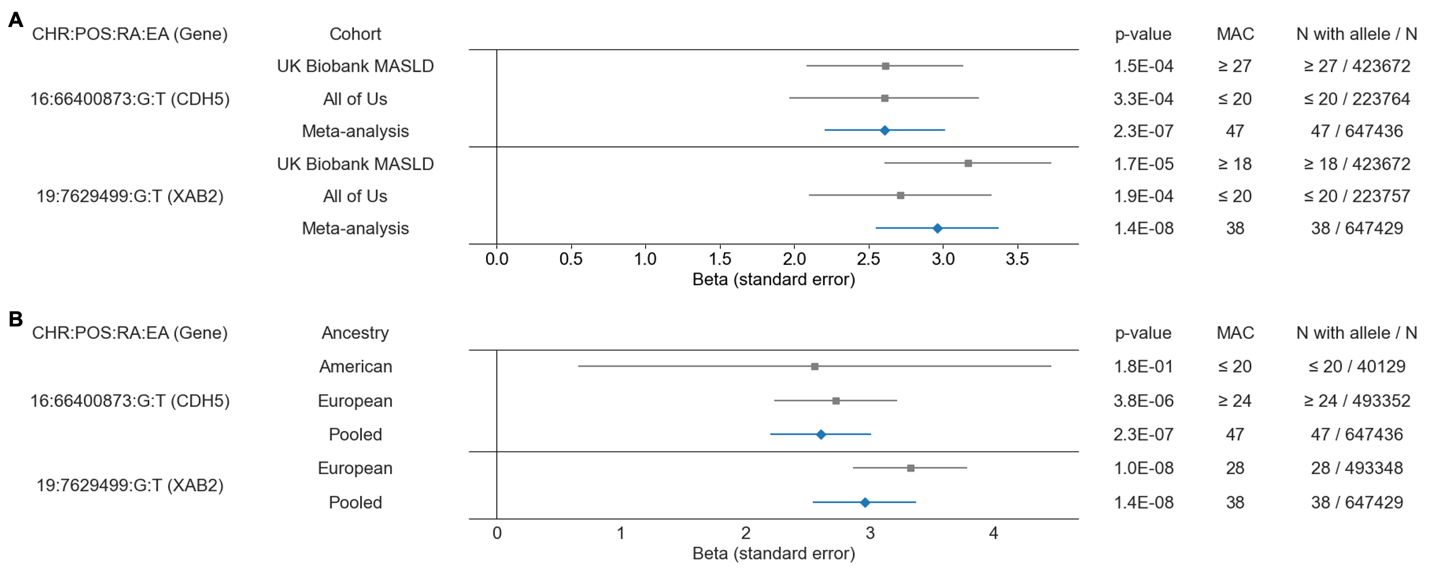


**(A)** Associations for pooled ancestry analyses within each cohort (grey squares) and for meta-analyses of these associations across cohorts (blue diamonds). Within each cohort, we performed association testing only for variants with a minor allele count (MAC) ≥ 10. Values with “≥” or “≤” indicate censoring to comply with *All of Us* policies. **(B)** Associations for ancestry-stratified meta-analyses across cohorts (grey squares) and for pooled ancestry meta-analyses across cohorts (blue diamonds; same as **A**). Within each cohort, we performed association testing only for variants with a MAC ≥ 5 within a specific ancestry. For all meta-analyses, we obtained beta and standard error estimates from an effect size and standard error-based meta-analysis and p-values from a sample size and direction of effect-based meta-analysis. Error bars represent standard errors. Complete data is available in **Additional file 1: Table S4**.

**Fig. S3: Cohort-stratified, ancestry-stratified, and pooled gene-level associations for true phenotypes**


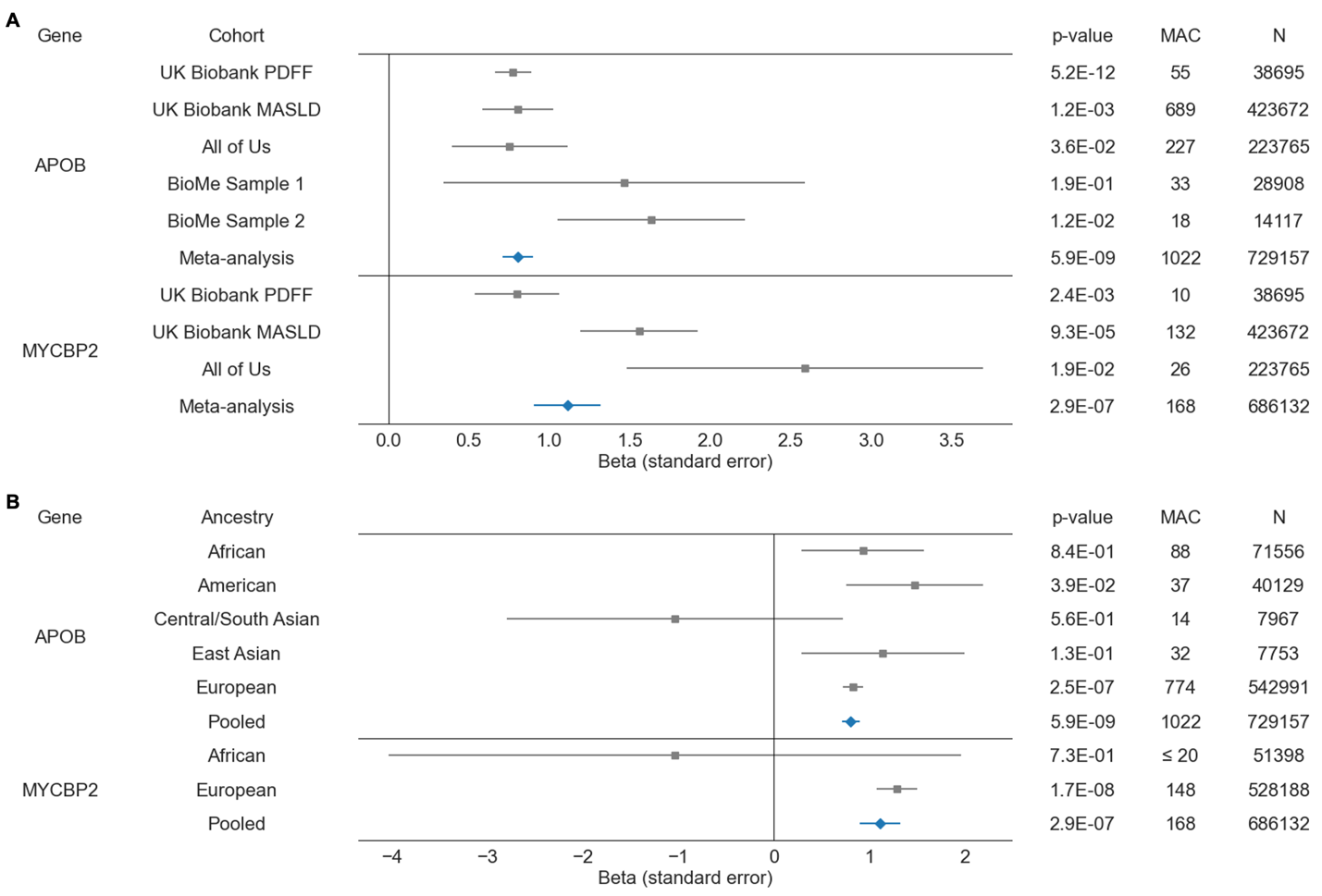


**(A)** Associations for pooled ancestry analyses within each cohort (grey squares) and for meta-analyses of these associations across cohorts (blue diamonds). Within each cohort, we performed association testing only for genes with a minor allele count (MAC) ≥ 10. **(B)** Associations for ancestry-stratified meta-analyses across cohorts (grey squares) and for pooled ancestry meta-analyses across cohorts (blue diamonds; same as **A**). Within each cohort, we performed association testing only for genes with a MAC ≥ 5 within a specific ancestry. The value with “≤” indicates censoring to comply with *All of Us* policies. For all meta-analyses, we obtained beta and standard error estimates from an effect size and standard error-based meta-analysis and p-values from a sample size and direction of effect-based meta-analysis. Error bars represent standard errors. Complete data is available in **Additional file 1: Table S6**.

**Fig. S4: Comparison of true and predicted phenotype effects for 40 previously reported common variants**

Shaded areas around regression lines represent 95% confidence intervals. Error bars represent standard errors (SE).

**Fig. S5: Quantile-quantile plots for predicted phenotype single variant associations**

**
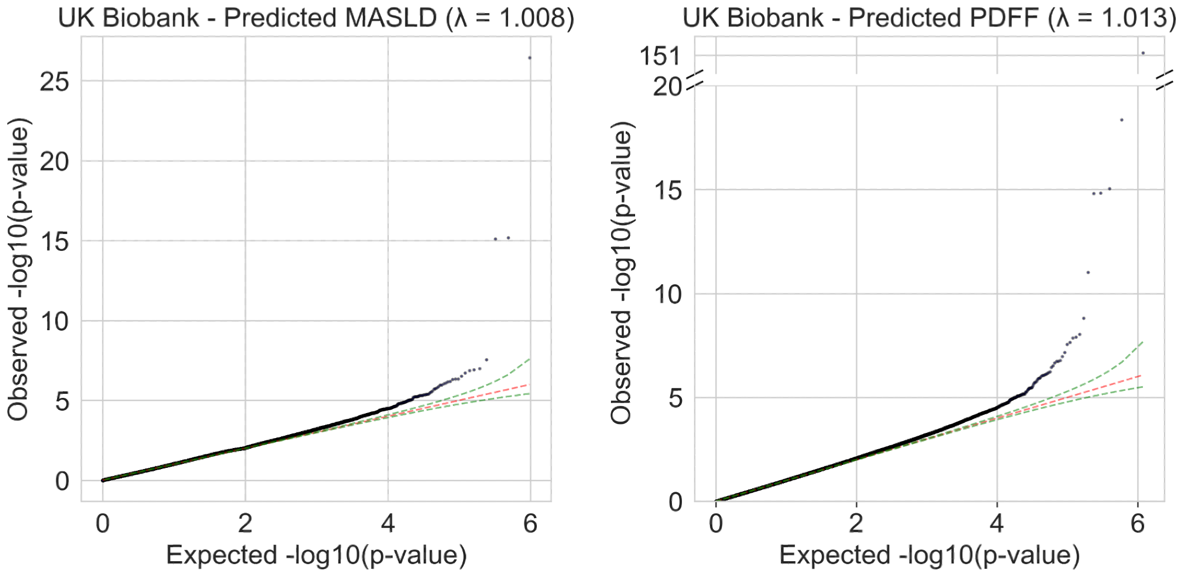
**

Green dashed lines represent the 95% confidence interval for expected -log_10_(p-values).

**Fig. S6: Comparison of true and predicted phenotype effects for variants identified by predicted phenotypes**

**
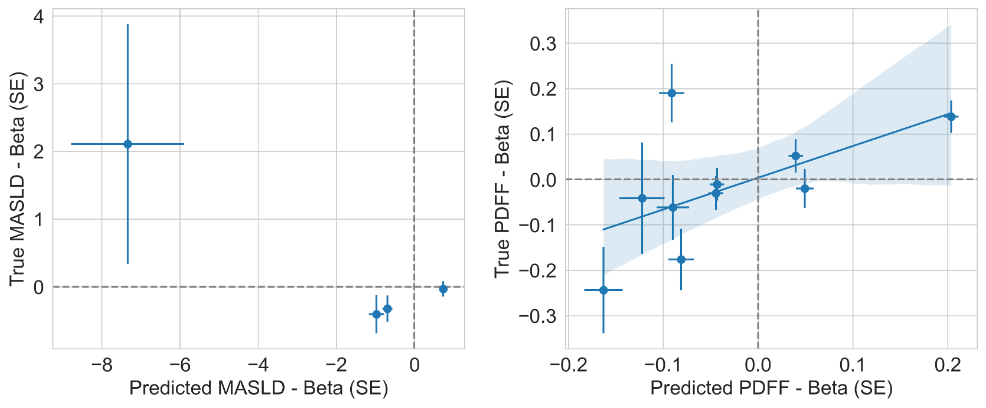
**

The shaded area around the regression line represents a 95% confidence interval. Error bars represent standard errors (SE).

**Fig. S7: Ancestry-stratified and pooled single variant associations for predicted phenotypes**

**
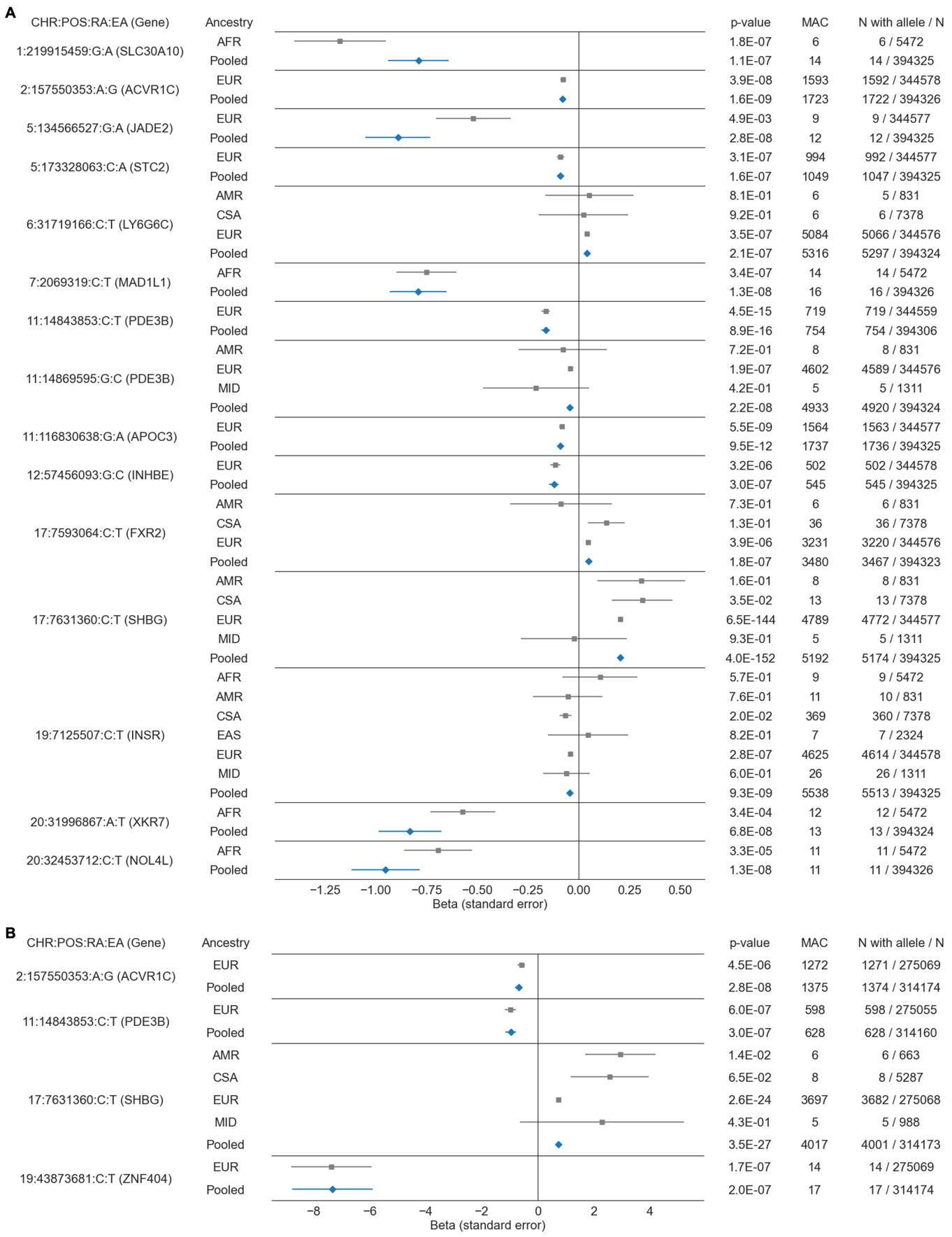
**

**(A)** Associations for predicted PDFF. **(B)** Associations for predicted MASLD. Grey squares represent ancestry-stratified associations while blue diamonds represent pooled ancestry associations. For pooled ancestry analyses, we performed association testing only for variants with a minor allele count (MAC) ≥ 10, while for ancestry-stratified analyses, we performed association testing only for variants with a MAC ≥ 5 within a specific ancestry. Error bars represent standard errors. Complete data is available in **Additional file 1:** **Table S21**. **Abbreviations:** AFR (African), AMR (American), CSA (Central/South Asian), EAS (East Asian), EUR (European), MID (Middle Eastern).

**Fig. S8: Ancestry-stratified and pooled gene-level associations for predicted phenotypes**

**
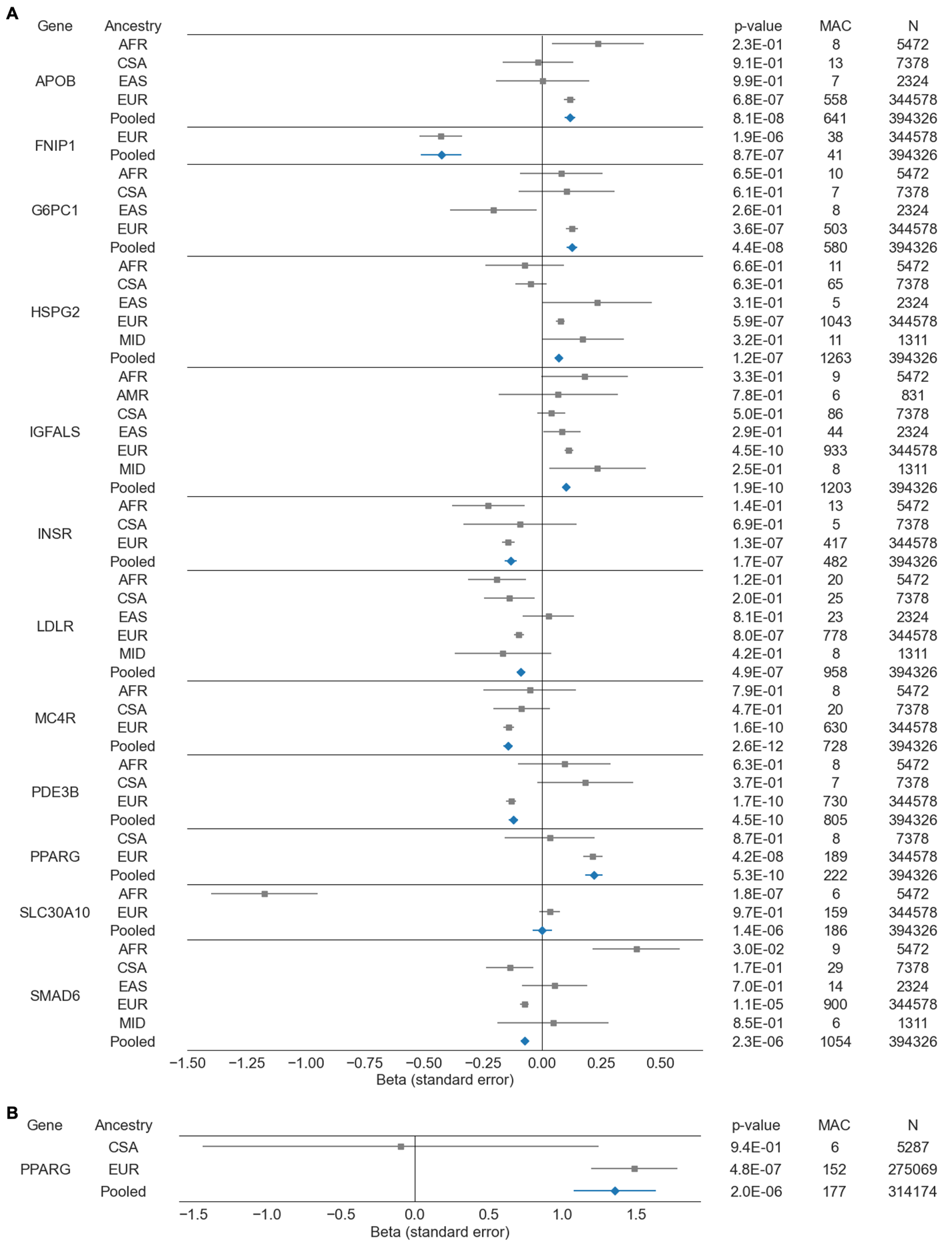
**

**(A)** Associations for predicted PDFF. **(B)** Associations for predicted MASLD. Grey squares represent ancestry-stratified associations while blue diamonds represent pooled ancestry associations. For pooled ancestry analyses, we performed association testing only for genes with a minor allele count (MAC) ≥ 10, while for ancestry-stratified analyses, we performed association testing only for genes with a MAC ≥ 5 within a specific ancestry. Error bars represent standard errors. Complete data is available in **Additional file 1:** **Table S21**. **Abbreviations:** AFR (African), AMR (American), CSA (Central/South Asian), EAS (East Asian), EUR (European), MID (Middle Eastern).
